# Association of Direct Oral Anticoagulation Management Strategies With Clinical Outcomes for Adults With Atrial Fibrillation

**DOI:** 10.1001/jamanetworkopen.2023.21971

**Published:** 2023-07-06

**Authors:** Catherine G. Derington, Glenn K. Goodrich, Stanley Xu, Nathan P. Clark, Kristi Reynolds, Jaejin An, Daniel M. Witt, David H. Smith, Maureen O’Keeffe-Rosetti, Daniel T. Lang, P. Michael Ho, T. Craig Cheetham, Angela C. Comer, Jordan B. King

**Affiliations:** 1 Intermountain Healthcare Department of Population Health Sciences, Spencer Fox Eccles University of Utah School of Medicine, Salt Lake City; 2 Institute for Health Research, Kaiser Permanente Colorado, Denver; 3 Department of Research and Evaluation, Kaiser Permanente Southern California, Pasadena; 4 Department of Pharmacy, Kaiser Permanente Colorado, Aurora; 5 Department of Pharmacotherapy, University of Utah College of Pharmacy, Salt Lake City; 6 Center for Health Research, Kaiser Permanente Northwest, Portland, Oregon; 7 Southern California Permanente Medical Group, Los Angeles; 8 Division of Cardiology, University of Colorado Anschutz Medical Campus, Aurora; 9 Cardiology Section, Veterans Affairs Eastern Colorado Health Care System, Aurora; 10 Chapman University School of Pharmacy, Irvine, California

## Abstract

**Importance:**

Anticoagulation management services (AMSs; ie, warfarin clinics) have evolved to include patients treated with direct oral anticoagulants (DOACs), but it is unknown whether DOAC therapy management services improve outcomes for patients with atrial fibrillation (AF).

**Objective:**

To compare outcomes associated with 3 DOAC care models for preventing adverse anticoagulation-related outcomes among patients with AF.

**Design, Setting, and Participants:**

This retrospective cohort study included 44 746 adult patients with a diagnosis of AF who initiated oral anticoagulation (DOAC or warfarin) between August 1, 2016, and December 31, 2019, in 3 Kaiser Permanente (KP) regions. Statistical analysis was conducted from August 2021 through May 2023.

**Exposures:**

Each KP region used an AMS to manage warfarin but used distinct approaches to DOAC care: (1) usual care (UC) by the prescribing clinician, (2) UC plus an automated population management tool (PMT), or (3) pharmacist-managed AMS care. Propensity scores and inverse probability of treatment weights (IPTWs) were estimated. Direct oral anticoagulant care models were first indirectly compared using warfarin as a common comparator within each region and then directly compared across regions.

**Main Outcomes and Measures:**

Patients were followed up until the first occurrence of an outcome (composite of thromboembolic stroke, intracranial hemorrhage, other major bleeding, or death), discontinuation of KP membership, or December 31, 2020.

**Results:**

Overall, 44 746 patients were included: 6182 in the UC care model (3297 DOAC; 2885 warfarin), 33 625 in the UC plus PMT care model (21 891 DOAC; 11 734 warfarin), and 4939 in the AMS care model (2089 DOAC; 2850 warfarin). Baseline characteristics (mean [SD] age, 73.1 [10.6] years, 56.1% male, 67.2% non-Hispanic White, median CHA_2_DS_2_-VASc [congestive heart failure, hypertension, age ≥75 years, diabetes, stroke, vascular disease, age 65-74 years, female sex] score of 3 [IQR, 2-5]) were well balanced after IPTW. Over a median follow-up of 2 years, patients who received the UC plus PMT or AMS care model did not have significantly better outcomes than those who received UC. The incidence rate of the composite outcome was 5.4% per year for DOAC and 9.1% per year for warfarin for those in the UC group, 6.1% per year for DOAC and 10.5% per year for those in the UC plus PMT group, and 5.1% per year for DOAC and 8.0% per year for those in the AMS group. The IPTW-adjusted hazard ratios (HRs) for the composite outcome comparing DOAC vs warfarin were 0.91 (95% CI, 0.79-1.05) in the UC group, 0.85 (95% CI, 0.79-0.90) in the UC plus PMT group, and 0.84 (95% CI, 0.72-0.99) in the AMS group (*P* = .62 for heterogeneity across care models). When directly comparing patients receiving DOAC, the IPTW-adjusted HR was 1.06 (95% CI, 0.85-1.34) for the UC plus PMT group vs the UC group and 0.85 (95% CI, 0.71-1.02) for the AMS group vs the UC group.

**Conclusions and Relevance:**

This cohort study did not find appreciably better outcomes for patients receiving DOAC who were managed by either a UC plus PMT or AMS care model compared with UC.

## Key Points

**Question:**

Are system-level direct oral anticoagulant (DOAC) therapy management services associated with anticoagulation-related outcomes for patients with atrial fibrillation (AF) relative to usual care?

**Findings:**

This cohort study evaluated 44 746 patients with AF initiating DOACs (managed using 2 different system-level care models or usual care) and warfarin therapy (managed by warfarin clinics). Patients receiving DOACs vs warfarin were less likely to experience adverse outcomes overall, but neither system-level DOAC management service was appreciably superior to usual DOAC care.

**Meaning:**

Randomized trials are needed to confirm the findings of this study, which suggest that there is no effectiveness advantage to system-level DOAC therapy management services vs usual care for patients with AF.

## Teaser

This cohort study compares outcomes associated with 3 direct oral anticoagulant care (DOAC) models for preventing adverse anticoagulation-related outcomes among patients with atrial fibrillation.

## Introduction

Despite debate in anticoagulation stewardship circles for the past decade, the question as to whether direct oral anticoagulant (DOAC) stewardship programs are needed to improve major anticoagulation-related adverse events remains unsatisfactorily answered. Anticoagulants are consistently identified as one of the most common sources of preventable adverse drug events in health care settings,^1,2,3,4,5^ and DOACs now account for approximately 2 in 3 oral anticoagulant prescriptions among patients with atrial fibrillation (AF).^6,7^ Although DOACs have a favorable safety profile compared with warfarin, anticoagulants—including DOACs—are inherently dangerous medications. Many health care systems now take a proactive role in preventing adverse DOAC outcomes by enrolling patients in the same services originally developed to manage patients taking warfarin.^8^ However, there are substantial differences between clinical management of DOACs and warfarin, and there is little evidence to demonstrate that DOAC management services improve outcomes such as stroke and bleeding.

Kaiser Permanente (KP) is one of the nation’s largest not-for-profit health plans, providing care across 8 distinct regions. Each KP region has its own local leadership, autonomy, and flexibility to establish health services. This autonomy, coupled with a paucity of evidence on how to best care for patients using DOACs, resulted in substantially different approaches to DOAC management across KP regions. We therefore conducted a pharmacoepidemiologic study to assess whether the rates of anticoagulation-related outcomes (ie, bleeding, stroke, and death) among patients initiating DOACs varied by the distinct DOAC care models used.

## Methods

### Study Setting

This retrospective cohort study used clinical and administrative data from August 1, 2016, to December 31, 2019, from 3 KP regions: Northwest (KPNW), Southern California (KPSC), and Colorado (KPCO). Each region uses an electronic health record (EHR) system (Epic Systems) to document and store health information, which is then loaded into a virtual data warehouse, a repository used for clinical research (eMethods in Supplement 1).^9^ This study was reviewed and approved by the KPCO institutional review board with a waiver of informed consent owing to the retrospective nature of this study; the KPNW and KPSC institutional review boards reviewed and ceded oversight to KPCO. This study adheres to the Strengthening the Reporting of Observational Studies in Epidemiology (STROBE) reporting guideline for cohort studies (eTable 1 in Supplement 1).^10^

### Anticoagulation Management Care Models

The approaches to managing anticoagulation therapy at KP, including the services and clinical interventions provided, are detailed in the eMethods in Supplement 1 and elsewhere.^11,12,13^ Briefly, all regions use a centralized anticoagulation management service (AMS), commonly referred to as warfarin clinics, to manage patients receiving warfarin therapy. Kaiser Permanente also uses a national anticoagulation quality improvement work group with representation from each KP region to track anticoagulation-related quality metrics, including warfarin time in therapeutic range. Tracking these internal metrics has found minimal differences in time in therapeutic range across regions, with all regions reporting a time in therapeutic range greater than 70%.

We assessed 3 mutually exclusive DOAC management models: (1) usual care (UC), (2) UC plus an automated population management tool (PMT), and (3) AMS. Usual care refers to the absence of a standardized, system-level DOAC management service. In the UC plus PMT model, the prescribing clinician managed all aspects of DOAC use as described above in the UC model. In addition, 3 clinical pharmacists who have specialized training in anticoagulation management received weekly reports from an automated PMT in the EHR, which identified patients who may have a DOAC-related medication problem based on dispensing and laboratory inputs pulled from the EHR. Finally, in the AMS model, patients who were prescribed a DOAC were proactively enrolled in the same pharmacist-led AMS that manages warfarin therapy. Patients who were not initially referred to the AMS by their prescribing clinician were identified for potential enrollment using electronic prescription and pharmacy dispensing data.

### Analytic Approach

We chose a priori to conduct a cohort study within each participating KP region comparing the initiation of a DOAC (ie, dabigatran, rivaroxaban, apixaban, or edoxaban) vs initiation of warfarin among patients with AF (indirect comparison). Based on peer review feedback, we also conducted a post hoc analysis in which we directly compared the DOAC groups across participating KP regions (direct comparison). For a more detailed discussion of the rationale for our analytic approach, see the eMethods in Supplement 1.

We used pharmacy dispensing records from KP pharmacies to identify patients initiating a medication of interest between August 1, 2016, and December 31, 2019, and created new-user cohorts that were mutually exclusive within and between regions, with the date of the patient’s first dispensed oral anticoagulant defining his or her index date (eFigure 1 in Supplement 1). We excluded patients who (1) had a history of oral anticoagulant use in the past 6 months (ie, new-user design), (2) were dispensed more than 1 oral anticoagulant (ie, 2 DOACs or a DOAC and warfarin) on the index date, (3) died prior to the index date, (4) were younger than 18 years of age on the index date, (5) had less than 365 days of KP health plan membership prior to the index date (lapses of <45 days were allowed), or (6) did not have a diagnosis of AF within 1 year before or 7 days after the index date. Patients were censored at an outcome of interest, loss of KP membership, or December 31, 2020, whichever occurred first.

### Outcomes

The primary outcome for this study was a “net clinical benefit” composite of thromboembolic stroke, major bleeding, or death (see eTable 2 in Supplement 1 for definitions). Each of the components of the composite outcome were also assessed individually as secondary outcomes. In addition, we evaluated major bleeding as intracranial hemorrhage, gastrointestinal bleeding, or extracranial major bleeding. We defined each outcome according to validated claims-based algorithms from the Food and Drug Administration’s Sentinel Initiative.^14,15^ In addition, we explored the adherence of patients treated with DOACs using the proportion of days’ covered metric and persistence at 12 months (eMethods in Supplement 1).

### Covariates

Using data from the EHR and administrative claims, we identified patient-level demographic characteristics (eg, age and sex), census tract–level income and educational attainment, comorbidities (eg, diabetes), ambulatory blood pressure values (ie, systolic and diastolic blood pressure), laboratory values (eg, serum creatinine), and recent health care encounters (eg, hospitalizations) within the 6 to 12 months prior to the index date (see eTable 2 in Supplement 1 for definitions). We used administrative and clinical data to calculate the CHA_2_DS_2_-VASc (congestive heart failure, hypertension, age ≥75 years, diabetes, stroke, vascular disease, age 65-74 years, female sex) score and ATRIA (Anticoagulation and Risk Factors in Atrial Fibrillation) stroke and bleeding risk scores to summarize patients’ thromboembolic stroke and major bleeding risks (eMethods in Supplement 1).^16,17,18^ We collected data on race and ethnicity because patients who identify as races and ethnicities other than non-Hispanic White are less likely to be prescribed DOACs and have worse anticoagulation-related outcomes.^19,20,21^

### Statistical Analysis

Statistical analysis was conducted from August 2021 through May 2023. Analyses were conducted for each DOAC vs warfarin cohort within each region, as well as DOAC vs DOAC cohorts across the regions. For missing continuous variables (eTable 3 in Supplement 1), we used a linear regression approach with available factors as covariates in the SAS procedure PROC MI (SAS Institute Inc). For self-reported race and ethnicity, we imputed missing values using site-specific distributions of available race and ethnicity information. We estimated propensity scores (PSs) using logistic regression to model the probability of receiving the exposure of interest (either DOAC vs warfarin or a system-level DOAC care model vs DOAC UC) as a function of baseline patient characteristics (eMethods in Supplement 1) and used the PS to generate for each patient an inverse probability of treatment weight (IPTW). A candidate list of potential patient characteristics to be included in the PS models was determined a priori based on clinical knowledge and published literature of the characteristics likely to be associated with our composite outcome (eTable 4 in Supplement 1). We assessed covariate balance before and after IPTW using absolute standardized mean difference (ASMD), with a value of less than 0.1 considered balanced.^22,23^

Next, we estimated hazard ratios (HRs) for the association of exposure groups and the outcomes of interest using IPTW Cox proportional hazards regression. Bootstrapping with 250 replicates with replacement was used to generate the SEs for the 95% CIs. We assessed possible departures from the proportional hazards assumption by testing the interaction between treatment group and time, and none were detected. In the a priori comparison wherein DOAC groups were compared indirectly by using warfarin as a common comparator, we tested whether heterogeneity existed in the DOAC vs warfarin treatment effect across the 3 DOAC care models by running a Monte Carlo simulation with 30 000 repetitions using the β coefficient and SEs from the Cox proportional hazards regression models, assuming a normal distribution. We then tested for differences between the care models using a 1-way analysis of variance.

Only the indirect comparisons with warfarin, which were determined a priori, were repeated in subgroups according to age, sex, race and ethnicity, body weight, creatinine clearance, CHA_2_DS_2_-VASc score, and ATRIA bleeding score. In sensitivity analyses, we repeated analyses using several different covariate adjustment and PS application strategies (eMethods in Supplement 1). We also varied exclusion criteria and outcome assessment. All *P* values were from 2-sided tests and results were deemed statistically significant at *P* < .05. Analyses were conducted using SAS, version 9.4 (SAS Institute Inc).

## Results

### Patient Characteristics

Overall, 44 746 patients (mean [SD] age, 73.1 [10.6] years, 56.1% male, 67.2% non-Hispanic White, median CHA_2_DS_2_-VASc score of 3 [IQR, 2-5]) met our eligibility criteria and were included in this analysis: 6182 patients at KPNW (3297 DOAC and 2885 warfarin), 33 625 patients at KPSC (21 891 DOAC and 11 734 warfarin), and 4939 at KPCO (2089 DOAC and 2850 warfarin) (eFigure 2 in Supplement 1). The median length of patient follow-up was 2.2 years in KPNW, 2.0 years in KPSC, and 2.2 years in KPCO.

Compared with patients who initiated warfarin, those who initiated DOACs were more likely to be younger, male, non-Hispanic White, more than 60 kg in weight, and former or never smokers and have hypertension (Table 1; see eTable 5 in Supplement 1 for additional characteristics). The most common DOAC used in all regions was dabigatran (84%-93%). Compared with patients initiating DOACs receiving treatment in KPNW, patients initiating DOACs in KPSC and KPCO were less likely to be non-Hispanic White, have an annual income less than $50 000, and have a body mass index of 30 or more (calculated as weight in kilograms divided by height in meters squared). Patients initiating DOACs in KPSC and KPCO also had a lower creatinine clearance on average and were more likely to have a history of bleeding. After multiple imputation, PS generation, and application of IPTW, no meaningful differences in baseline covariates remained between patients treated with DOACs and patients treated with warfarin in all 3 regions, or among patients initiating DOACs between regions (ASMD, <0.1) (eFigures 3-6 in Supplement 1).

**Table 1.** Selected Characteristics of DOAC and Warfarin Users Across 3 DOAC Management Models, Before Weighting and Multiple Imputation^a^

Characteristic	UC		UC plus PMT		AMS	
	DOAC	Warfarin	DOAC	Warfarin	DOAC	Warfarin
No. of patients^b^	3297	2885	21 891	11 734	2089	2850
Dabigatran, No. (%)	3061 (92.8)	NA	20 044 (91.6)	NA	1749 (83.7)	NA
Apixaban, No. (%)	149 (4.5)	NA	1379 (6.3)	NA	251 (12.0)	NA
Rivaroxaban, No. (%)	83 (2.5)	NA	457 (2.1)	NA	86 (4.1)	NA
Year of index date, No. (%)^c^						
2016	225 (6.8)	567 (19.7)	1728 (7.9)	3883 (33.1)	190 (9.1)	543 (19.1)
2017	720 (21.8)	1074 (37.2)	5474 (25.0)	4441 (37.8)	524 (25.1)	998 (35.0)
2018	1004 (30.5)	776 (26.9)	6933 (31.7)	2062 (17.6)	606 (29.0)	748 (26.2)
2019	1348 (40.9)	468 (16.2)	7756 (35.4)	1348 (11.5)	769 (36.8)	561 (19.7)
Demographic characteristics, No. (%)						
Age, y	71.3 (10.6)	73.9 (10.1)	72.2 (11.1)	74.2 (10.9)	72.5 (10.5)	74.6 (10.3)
Sex						
Male	1836 (55.7)	1562 (54.1)	12 431 (56.8)	6602 (56.3)	1172 (56.1)	1509 (52.9)
Female	1461 (44.3)	1322 (45.8)	9459 (43.2)	5132 (43.7)	917 (43.9)	1341 (47.1)
Non-Hispanic White	3027 (91.8)	2604 (90.3)	13 548 (61.9)	6732 (57.4)	1776 (85.0)	2366 (83.0)
Lives in a census tract where						
>20% of Residents have less than a high school degree	386 (11.7)	330 (11.4)	6002 (27.4)	3659 (31.2)	206 (9.9)	367 (12.9)
Annual household income is <$50 000 USD	1097 (33.3)	968 (33.6)	3448 (15.8)	2110 (18.0)	416 (19.9)	679 (23.8)
Current tobacco use	131 (4.0)	132 (4.6)	722 (3.3)	339 (2.9)	92 (4.4)	136 (4.8)
Physiological variables						
Weight, mean (SD), kg	91.6 (24.1)	92.3 (26.5)	87.0 (24.0)	84.8 (24.2)	85.4 (22.2)	85.6 (23.5)
BMI, mean (SD)	31.1 (7.3)	31.7 (8.4)	29.8 (7.0)	29.5 (7.2)	28.9 (6.5)	29.6 (7.1)
Creatinine clearance, mL/min, mean (SD)	91.7 (42.1)	81.4 (43.7)	84.4 (39.4)	70.1 (41.2)	80.5 (33.3)	75.7 (37.4)
AST, U/L, mean (SD)	33.7 (23.8)	36.2 (95.5)	30.6 (40.5)	32.3 (51.3)	34.3 (76.3)	38.4 (180.5)
ALT, U/L, mean (SD)	33.1 (30)	35.7 (102.3)	26.4 (30.6)	26.7 (45.6)	33.4 (60.9)	36.2 (114.5)
Stroke and bleeding risk scores, median (IQR)						
CHA_2_DS_2_-VASc	3 (2-5)	4 (3-5)	4 (2-5)	4 (3-5)	3 (2-5)	4 (3-5)
ATRIA stroke risk	6 (5-8)	7 (5-9)	7 (5-8)	8 (6-9)	7 (5-8)	7 (5-8)
ATRIA bleeding risk	2 (1-3)	3 (1-5)	2 (1-3)	3 (1-6)	2 (1-3)	3 (1-4)
Medical conditions, No. (%)						
Type of AF or flutter						
Atrial flutter	30 (0.9)	8 (0.3)	107 (0.5)	28 (0.2)	47 (2.3)	30 (1.1)
Paroxysmal	1377 (41.8)	1066 (36.9)	9843 (45.0)	4605 (39.2)	1025 (49.1)	1178 (41.3)
Persistent	115 (3.5)	107 (3.7)	673 (3.1)	341 (2.9)	120 (5.7)	103 (3.6)
Chronic	286 (8.7)	256 (8.9)	944 (4.3)	914 (7.8)	119 (5.7)	177 (6.2)
Unspecified or unknown	1489 (45.2)	1448 (50.2)	10 324 (47.2)	5846 (49.8)	778 (37.2)	1362 (47.8)
Alcohol abuse	136 (4.1)	97 (3.4)	976 (4.5)	486 (4.1)	81 (3.9)	83 (2.9)
History of thromboembolic stroke	179 (5.4)	155 (5.4)	1055 (4.8)	442 (3.8)	153 (7.3)	173 (6.1)
History of GI bleeding	6 (0.2)	16 (0.6)	87 (0.4)	91 (0.8)	19 (0.9)	42 (1.5)
History of intracranial bleeding	8 (0.2)	7 (0.2)	98 (0.4)	61 (0.5)	16 (0.8)	27 (0.9)
History of extracranial major bleeding	8 (0.2)	19 (0.7)	97 (0.4)	101 (0.9)	19 (0.9)	44 (1.5)
Diabetes	872 (26.4)	1053 (36.5)	7354 (33.6)	5113 (43.6)	506 (24.2)	844 (29.6)
Heart failure	845 (25.6)	977 (33.9)	5559 (25.4)	4900 (41.8)	568 (27.2)	997 (35.0)
Hypertension	2279 (69.1)	2113 (73.2)	17 040 (77.8)	10 011 (85.3)	1393 (66.7)	2114 (74.2)
History of myocardial infarction	258 (7.8)	245 (8.5)	1140 (5.2)	901 (7.7)	134 (6.4)	151 (5.3)
Peripheral artery disease	322 (9.8)	368 (12.8)	1637 (7.5)	1395 (11.9)	199 (9.5)	310 (10.9)
Moderate-to-severe liver disease	29 (0.9)	44 (1.5)	126 (0.6)	142 (1.2)	14 (0.7)	32 (1.1)
Moderate-to-severe renal disease	838 (25.4)	1086 (37.6)	6305 (28.8)	5746 (49.0)	656 (31.4)	1272 (44.6)
Healthcare encounters during preindex period, median (IQR)						
Ambulatory care visits	9 (5-15)	10 (5-18)	10 (5-17)	13 (7-21)	7 (4-11)	7 (4-11)
Emergency department visits	1 (0-2)	1 (0-2)	1 (0-2)	1 (0-2)	0 (0-1)	0 (0-1)
Hospitalizations	0 (0-1)	1 (0-1)	0 (0-1)	1 (0-1)	0 (0-1)	1 (0-1)

Abbreviations: AF, atrial fibrillation; ALT, alanine aminotransferase; AMS, anticoagulation management service; AST, aspartate aminotransferase; ATRIA, Anticoagulation and Risk Factors in Atrial Fibrillation; BMI, body mass index (calculated as weight in kilograms divided by height in meters squared); CHA_2_DS_2_-VASc, congestive heart failure, hypertension, age ≥75 years, diabetes, stroke, vascular disease, age 65-74 years, female sex; DOAC, direct oral anticoagulant; GI, gastrointestinal; NA, not applicable; PMT, population management tool; UC, usual care; USD, US dollars.

SI conversion factors: To convert total ALT and AST to microkatals per liter, multiply by 0.0167.

^a^ Complete set of characteristics available in eTable 4 in Supplement 1.

^b^ Does not add up to 100% in each region because some patients were receiving multiple DOACs (UC, 4; UC plus PMT, 9; AMS, 3).

^c^ The index date was the date of first DOAC pharmacy fill; all values were collected in the 1 year prior to the index date.

### Indirect Comparison of the DOAC Care Models Using Warfarin as a Common Comparator Within Sites

**Figure 1.**
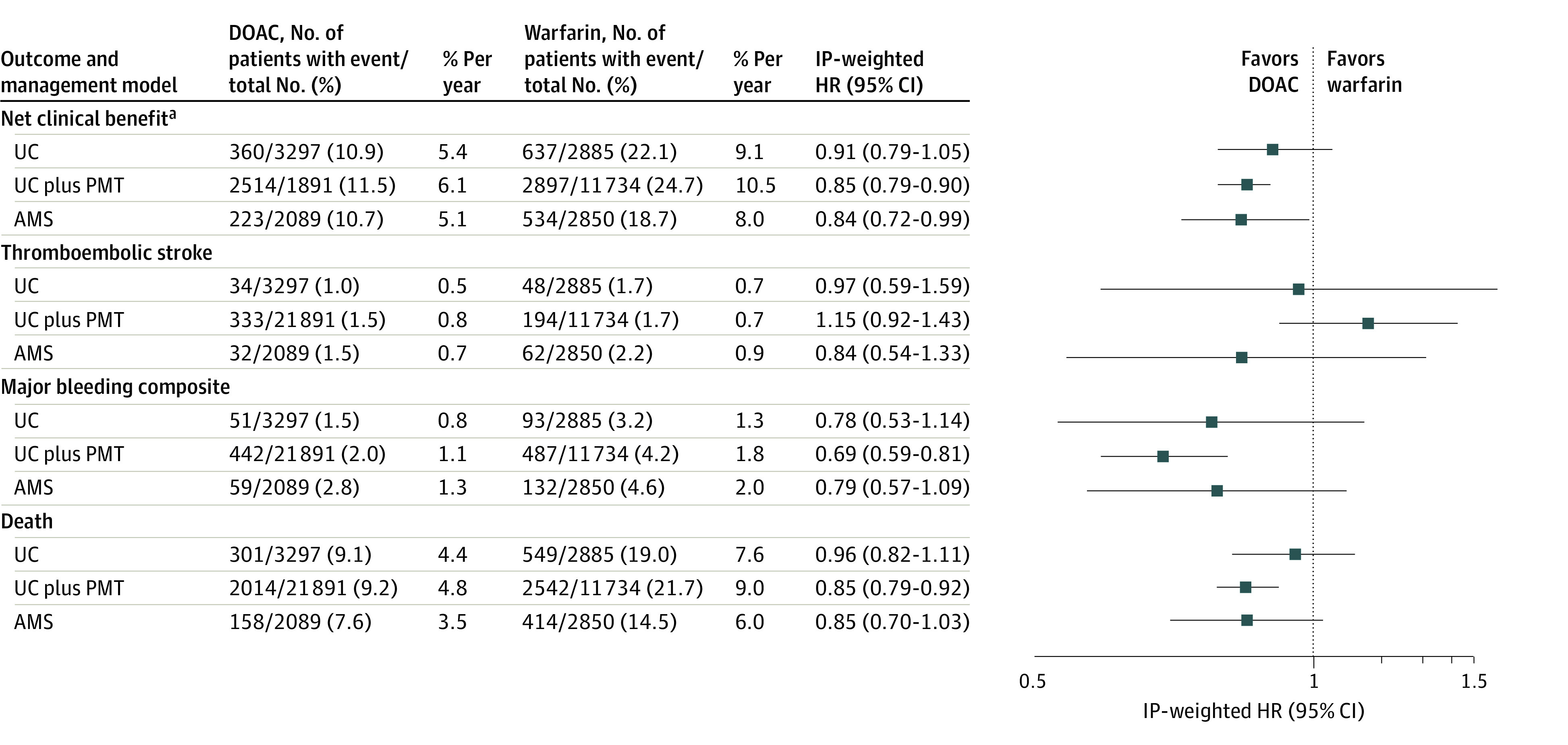
Association of Direct Oral Anticoagulant (DOAC) vs Warfarin Use With Major Clinical Outcomes by DOAC Management Model Event rates are derived from each site independently and are unweighted; hazard ratios (HRs) are weighted by the inverse of the propensity score. AMS indicates anticoagulation management service; IP, inverse probability; PMT, population management tool; and UC, usual care. ^a^Composite end point of thromboembolic stroke, intracranial hemorrhage, gastrointestinal bleeding, extracranial major bleeding, or death.

**Figure 2.**
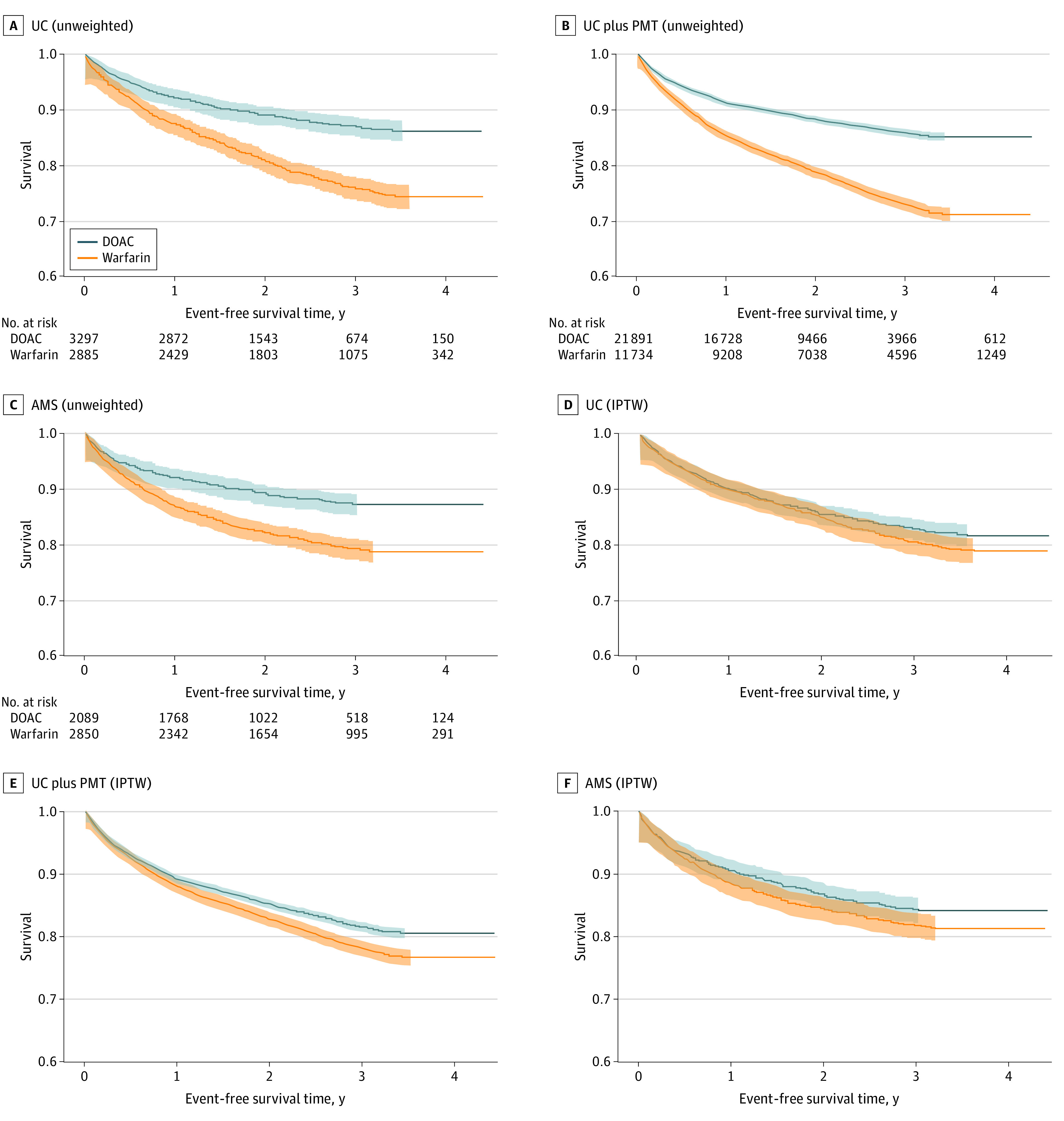
Kaplan-Meier Graph for the Association of Direct Oral Anticoagulant (DOAC) vs Warfarin Use With the Net Clinical Benefit Outcome by DOAC Care Model A-C, Unweighted curves for the net clinical benefit outcome in each DOAC care model. D-F, Weighted curves for the net clinical benefit outcome generated from inverse probability-weighted Cox proportional hazards regression models. The composite outcome was thromboembolic stroke, intracranial hemorrhage, gastrointestinal bleeding, extracranial major bleeding, or death. Patients were censored at the first occurrence of an outcome, loss of Kaiser Permanente membership, or December 31, 2020. Shaded regions around each line indicate the 95% CIs. Numbers at risk for the weighted analyses are omitted because the curves were generated from a model using weighted samples. AMS indicates anticoagulation management service; IPTW, inverse probability of treatment weighted; PMT, population management tool; and UC, usual care.

Over a median follow-up of 2 years, the incidence rate of the composite outcome was 5.4% per year for DOAC and 9.1% per year for warfarin for those in the UC group, 6.1% per year for DOAC and 10.5% per year for those in the UC plus PMT group, and 5.1% per year for DOAC and 8.0% per year for those in the AMS group (Figure 1). Initiation of DOACs in the UC model was not significantly associated with the net clinical benefit outcome compared with warfarin initiation (HR, 0.91; 95% CI, 0.79-1.05) (Figure 1). Direct oral anticoagulant therapy was associated with a lower incidence of the net clinical benefit outcome compared with warfarin in the UC plus PMT care model (HR, 0.85; 95% CI, 0.79-0.90) and AMS care model (HR, 0.84; 95% CI, 0.72-0.99) (*P* = .62 for heterogeneity across care models). The DOAC vs warfarin comparisons for the individual components of the composite outcome are shown in Figure 1, with only major bleeding (HR, 0.69; 95% CI, 0.59-0.81) and all-cause death (HR, 0.85; 95% CI, 0.79-0.92) in the UC plus PMT model reaching statistical significance. Kaplan-Meier graphs for the 3 DOAC management models are shown in Figure 2. When assessing each type of major bleeding individually, there was a significant reduction in intracranial hemorrhage with DOAC vs warfarin treatment in all 3 care models, and a significant reduction in extracranial major bleeding (HR, 0.79; 95% CI, 0.65-0.97) in the UC plus PMT care model (eFigure 7 in Supplement 1). There was no appreciable heterogeneity in the treatment effect of DOAC vs warfarin for the net clinical benefit outcome across DOAC care models or for any secondary outcomes.

### Direct Comparison of the 3 DOAC Management Models

**Figure 3.**
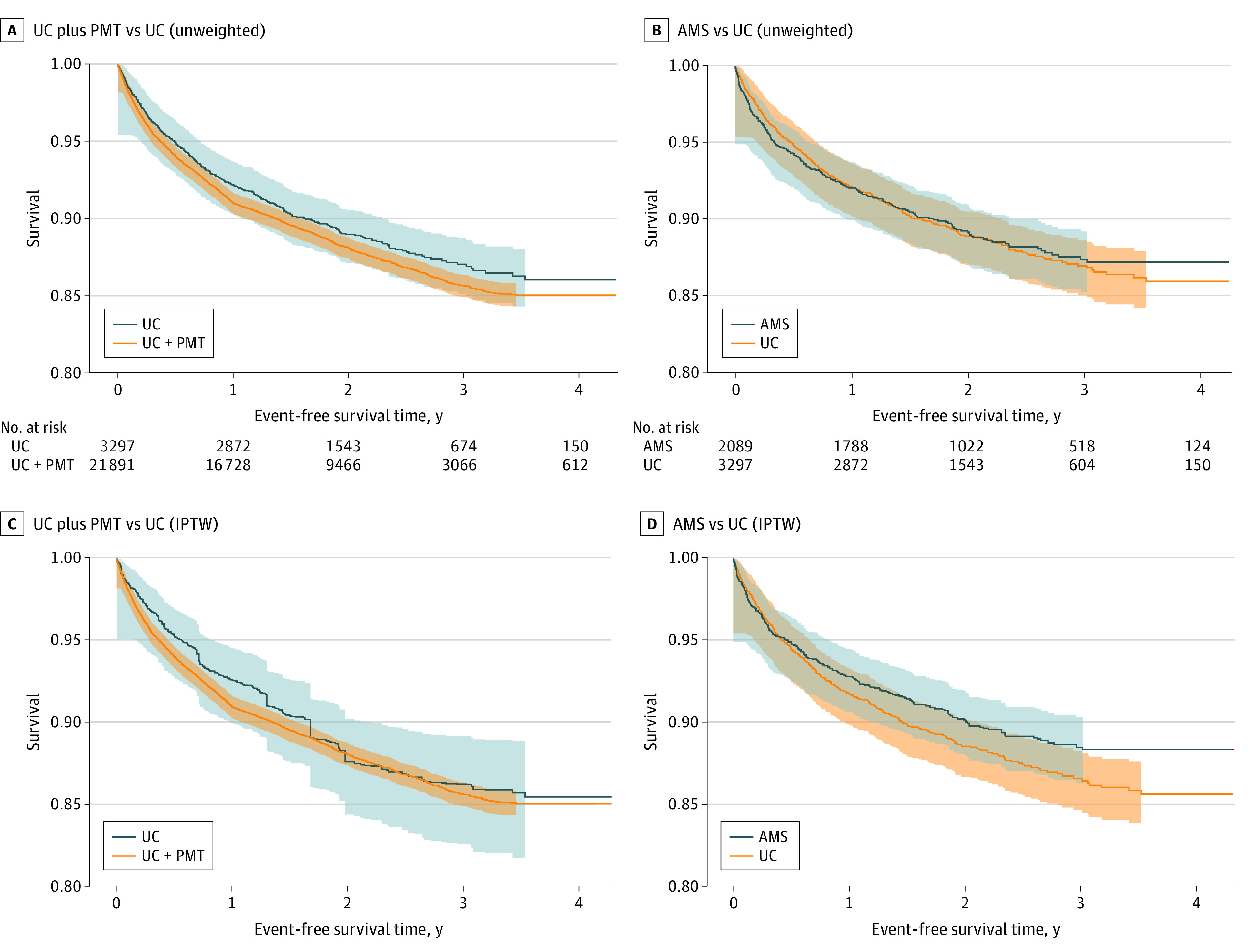
Kaplan-Meier Graph for the Association of Direct Oral Anticoagulant (DOAC) Use Between Care Models With the Net Clinical Benefit Outcome A and B, Unweighted curves for the net clinical benefit outcome between DOAC users in the usual care (UC) plus population management tool (PMT) vs UC care models, and anticoagulation management service (AMS) vs UC care models, respectively. C and D, Weighted curves for the net clinical benefit outcome generated from inverse probability-weighted Cox proportional hazards regression models. The composite outcome was thromboembolic stroke, intracranial hemorrhage, gastrointestinal bleeding, extracranial major bleeding, or death. Patients were censored at the first occurrence of an outcome, loss of Kaiser Permanente membership, or December 31, 2020. Shaded regions around each line indicate the 95% CIs. Numbers at risk for the weighted analyses are omitted because the curves were generated from a model using weighted samples. IPTW indicates inverse probability of treatment weighted.

There were no significant differences in the net clinical benefit outcome among patients initiating DOACs and receiving care in the UC plus PMT vs UC care models (HR, 1.06; 95% CI, 0.85-1.34) or AMS vs UC care models (HR, 0.85; 95% CI, 0.71-1.02) (Table 2). Patients initiating DOACs who received care in the AMS vs UC care model had a higher risk of major bleeding (HR, 1.53; 95% CI, 1.02-2.31) and a lower risk of death (HR, 0.70; 95% CI, 0.57-0.86). Kaplan-Meier graphs for the 3 DOAC management models are shown in Figure 3. There were no significant differences in other secondary outcomes, including the individual types of major bleeding, among patients initiating DOACs between the UC plus PMT vs UC care models or the AMS vs UC care models (eTable 6 in Supplement 1).

**Table 2.** Association of DOAC Management Models With Clinical Outcomes

Outcome	No. of total events during follow-up (% per year)^a^			IP-weighted hazard ratio (95% CI)	
	UC (n = 3297)	UC plus PMT (n = 21 891)	AMS (n = 2089)	UC plus PMT vs UC	AMS vs UC
Net clinical benefit^b^	360 (5.4)	2514 (6.1)	223 (5.1)	1.06 (0.85-1.34)	0.85 (0.71-1.02)
Thromboembolic stroke	34 (0.5)	333 (0.8)	32 (0.7)	1.70 (0.96-3.00)	1.30 (0.77-2.20)
Major bleeding^c^	51 (0.8)	442 (1.1)	59 (1.3)	1.26 (0.72-2.21)	1.53 (1.02-2.31)
All-cause death	301 (4.4)	2014 (4.8)	158 (3.5)	1.03 (0.80-1.33)	0.70 (0.57-0.86)

Abbreviations: AMS, anticoagulation management service; DOAC, direct oral anticoagulant; IP, inverse probability; PMT, population management tool; UC, usual care.

^a^ Unweighted.

^b^ Composite outcome of thromboembolic stroke, intracranial hemorrhage, gastrointestinal bleeding, extracranial major bleeding, and death.

^c^ Composite of intracranial hemorrhage, gastrointestinal bleeding, and extracranial major bleeding.

### Secondary, Subgroup, and Sensitivity Analyses

The proportion of patients initiating a DOAC with medication adherence of 80% or more at 12 months was 71.6% in the UC model, 68.6% in the UC plus PMT model, and 72.4% in the AMS care model (eTable 7 in Supplement 1). The proportion of patients initiating DOACs who continued receiving DOAC therapy at 12 months was 50.8% in the UC model, 49.7% in the UC plus PMT model, and 52.3% in the AMS care model (eFigure 8 in Supplement 1).

Results were similar among subgroups by age, sex, race and ethnicity, body weight, creatinine clearance, CHA_2_DS_2_-VASc score, and ATRIA bleeding score (eFigure 9 in Supplement 1). Results did not differ in sensitivity analyses varying the covariate adjustment method (eTable 8 in Supplement 1) or when including only complete cases, excluding patients with a prior history of events, assessing outcomes only in the first 90 days, or assessing outcomes after 90 days (eFigure 10 in Supplement 1).

## Discussion

Our goals with this study were to understand whether DOAC stewardship programs improve anticoagulation-related adverse outcomes and whether one program type was better than another. We observed that patients initiating a DOAC were significantly less likely than those initiating warfarin to experience a composite of thromboembolic stroke, intracranial hemorrhage, gastrointestinal bleeding, extracranial major bleeding, or death in the 2 regions that used system-level DOAC management services, but not in the region that used UC. However, this finding may be associated with sample size and statistical power, and we did not observe statistical heterogeneity in the DOAC vs warfarin treatment effects across the 3 regions. We did not observe appreciable differences in any outcomes when directly comparing the UC plus PMT and UC groups. The AMS group experienced more major bleeding but less all-cause death than the UC group. One-year DOAC adherence and persistence rates were similar in all 3 care models.

The motivation to implement health services for DOAC management is thus associated in part with several reports of inappropriate prescribing of and inadequate adherence with DOACs, important surrogate markers of major clinical outcomes. Single center studies have demonstrated high and widely varying estimates of inappropriate prescribing (5%-42%) depending on definitions used.^24,25,26^ Nationally, an estimated 13% of patients with AF receive a dosage regimen that is inconsistent with DOAC labeling recommendations—9.5% receive a dose lower than recommended and 3.4% receive a dose higher than recommended.^27^ Estimates of patients receiving concomitant medications with potential DOAC interactions range from 28% to 91%.^24,25,28^ Some have even argued that there is an “urgent need for expanding the traditional role of the anticoagulation clinic” to include DOAC care.^8^ In regions that used system-level DOAC management services in our analysis, DOAC therapy was significantly better than warfarin therapy and had outcomes similar to clinical trial outcomes.^29^ However, rationale for implementing system-level DOAC care is based on the assumption that real-world DOAC prescribing and adherence is less than ideal. In our analysis, this assumption did not hold true, as the region that used a UC approach had better than anticipated DOAC-related outcomes. What remains unknown is whether the outcomes achieved in the UC group are unique to KP (an integrated health system well known for high quality, carefully coordinated care) or represent UC across the US.

A persistent barrier to the widespread use of pharmacist-led AMS is cost, particularly given issues with reimbursement for pharmacist-delivered clinical services.^30^ A forthcoming cost-effectiveness analysis comparing the 3 DOAC management models will provide greater insight as to which model is most cost-effective, given the evidence from the current study that all 3 are similarly effective. Further research may also evaluate whether specific DOAC models could reduce health disparities in anticoagulation prescribing and outcomes, which persist in contemporary clinical care, as demonstrated by recent evidence from the Get With The Guidelines registry.^19^

### Strengths and Limitations

This study has some strengths, including a large population size from 3 regions of an integrated health system. The KP infrastructure allows for region-wide implementation of health services, eliminating the need for prescriber referrals and potential confounding by baseline risk. Each region has a standardized model that is implemented uniformly across the entire region, allowing for direct comparison of clinical outcomes between regions. The robust data infrastructure allowed for collection of several covariates for statistical adjustment, and we completed several sensitivity analyses to assess for robustness of results. Because KP is both the payer and provider of care, there are little to no missing data due to out-of-system use, particularly regarding inpatient care. Fewer than 5% of KP members fill their prescriptions from non-KP pharmacies, including when using low-cost generic medications.^31^

The findings of the current study should be interpreted within the context of the following known limitations. Because of benefit design, the most commonly used DOAC in all care models of the current analysis was dabigatran, which has a much lower share of the market nationally.^7,32^ It is possible that results may differ with a greater proportion of new users of other DOAC products (ie, apixaban, rivaroxaban) which have more frequent use nationally and in other health systems. The studied population was largely non-Hispanic White and of relatively high educational background. In the UC and UC plus PMT models, it is possible that clinician specialty or subspecialty was associated with results, although we were unable to assess this in our study. We used multiple imputation to address missing data, which assumes that the data are missing at random. However, multiple imputation can provide valuable insights for data that are missing not at random, and we performed several sensitivity analyses. Although our study had a significantly greater sample than previous reports, it is possible that our null finding is due to a lack of power to detect differences between the groups. Low outcome rates in subgroups should be interpreted with caution.

## Conclusions

Although safer and simpler to use than warfarin, the DOACs are high-risk medications. In this cohort study, DOACs were associated with favorable outcomes compared with warfarin, but we did not establish superiority of any system-level DOAC therapy management service over UC. The optimal approach to DOAC monitoring and management is unclear, and future randomized clinical trials are warranted.

## Notes

Supplement 1.
eMethods.
**eTable 1.** STROBE Reporting Checklist for Cohort Studies **eTable 2.** Variables Used to Define the Study Population, Baseline Covariates, Exposures, and Outcomes **eTable 3.** Missingness of Characteristics Used to Generate the Propensity Score, by KP Region **eTable 4.** Characteristics Included in the Final Propensity Score Model (DOAC vs Warfarin) for the a Priori Analysis **eTable 5.** Complete Set of Characteristics of DOAC and Warfarin Users Across Three DOAC Management Models, Before Weighting and Multiple Imputation **eTable 6.** Association of DOAC Management Models With Individual Bleeding Outcomes **eTable 7.** One-Year Medication Adherence Rates Among DOAC Users **eTable 8.** Hazard Ratios for the Primary Outcomes, by Covariate Adjustment Strategy (a Priori Analysis) **eFigure 1.** Study Design Schema **eFigure 2.** Flowchart for Patient Inclusion in the Current Study **eFigure 3.** Propensity Score Histograms for Being a DOAC vs Warfarin User (a Priori Analysis) **eFigure 4.** Balance of Patient Characteristics Before and After Inverse Probability of Treatment Weighting (a Priori Analysis) **eFigure 5.** Propensity Score Histograms for Receiving Care in a Specific DOAC Care Model Among DOAC Users (Post Hoc Analysis) **eFigure 6.** Balance of Patient Characteristics Before and After Inverse Probability of Treatment Weighting (Post Hoc Analysis) **eFigure 7.** Association of DOAC vs Warfarin Use and Bleeding Outcomes, by DOAC Management Model (a Priori Analysis) **eFigure 8.** Medication Persistence Among DOAC Users **eFigure 9.** Association of Incident DOAC vs Warfarin Use and the Composite Endpoint in Subgroups **eFigure 10.** Association of Incident DOAC vs Warfarin Use and the Composite Endpoint by Sensitivity Analysis
eReferences.

Supplement 2.
Data Sharing Statement
