# Supplementary material for: Association of Direct Oral Anticoagulation Management Strategies With Clinical Outcomes for Adults With Atrial Fibrillation
Source: JAMA Netw Open. 2023 Jul 6;6(7):e2321971. doi: 10.1001/jamanetworkopen.2023.21971 (PMC10326649; doi:10.1001/jamanetworkopen.2023.21971)
Supplement: Supplement 1. — eMethods. eTable 1. STROBE Reporting Checklist for Cohort Studies eTable 2. Variables Used to Define the Study Population, Baseline Covariates, Exposures, and Outcomes eTable 3. Missingness of Characteristics Used to Generate the Propensity Score, by KP Region eTable 4. Characteristics Included in the Final Propensity Score Model (DOAC vs Warfarin) for the a Priori Analysis eTable 5. Complete Set of Characteristics of DOAC and Warfarin Users Across Three DOAC Management Models, Before Weighting and Multiple Imputation eTable 6. Association of DOAC Management Models With Individual Bleeding Outcomes eTable 7. One-Year Medication Adherence Rates Among DOAC Users eTable 8. Hazard Ratios for the Primary Outcomes, by Covariate Adjustment Strategy (a Priori Analysis) eFigure 1. Study Design Schema eFigure 2. Flowchart for Patient Inclusion in the Current Study eFigure 3. Propensity Score Histograms for Being a DOAC vs Warfarin User (a Priori Analysis) eFigure 4. Balance of Patient Characteristics Before and After Inverse Probability of Treatment Weighting (a Priori Analysis) eFigure 5. Propensity Score Histograms for Receiving Care in a Specific DOAC Care Model Among DOAC Users (Post Hoc Analysis) eFigure 6. Balance of Patient Characteristics Before and After Inverse Probability of Treatment Weighting (Post Hoc Analysis) eFigure 7. Association of DOAC vs Warfarin Use and Bleeding Outcomes, by DOAC Management Model (a Priori Analysis) eFigure 8. Medication Persistence Among DOAC Users eFigure 9. Association of Incident DOAC vs Warfarin Use and the Composite Endpoint in Subgroups eFigure 10. Association of Incident DOAC vs Warfarin Use and the Composite Endpoint by Sensitivity Analysis eReferences. [file jamanetwopen-e2321971-s001.pdf]

Supplementary Online Content

Derington CG, Goodrich GK, Xu S, et al. Association of direct oral anticoagulation management strategies with clinical outcomes for adults with atrial fibrillation. *JAMA Netw Open.* 2023;6(7):e2321971. doi:10.1001/jamanetworkopen.2023.21971

eMethods.
eTable 1. STROBE Reporting Checklist for Cohort Studies
eTable 2. Variables Used to Define the Study Population, Baseline Covariates, Exposures, and Outcomes
eTable 3. Missingness of Characteristics Used to Generate the Propensity Score, by KP Region
eTable 4. Characteristics Included in the Final Propensity Score Model (DOAC vs Warfarin) for the a Priori Analysis
eTable 5. Complete Set of Characteristics of DOAC and Warfarin Users Across Three DOAC Management Models, Before Weighting and Multiple Imputation
eTable 6. Association of DOAC Management Models With Individual Bleeding Outcomes
eTable 7. One-Year Medication Adherence Rates Among DOAC Users
eTable 8. Hazard Ratios for the Primary Outcomes, by Covariate Adjustment Strategy (a Priori Analysis)
eFigure 1. Study Design Schema
eFigure 2. Flowchart for Patient Inclusion in the Current Study
eFigure 3. Propensity Score Histograms for Being a DOAC vs Warfarin User (a Priori Analysis)
eFigure 4. Balance of Patient Characteristics Before and After Inverse Probability of Treatment Weighting (a Priori Analysis)
eFigure 5. Propensity Score Histograms for Receiving Care in a Specific DOAC Care Model Among DOAC Users (Post Hoc Analysis)
eFigure 6. Balance of Patient Characteristics Before and After Inverse Probability of Treatment Weighting (Post Hoc Analysis)
eFigure 7. Association of DOAC vs Warfarin Use and Bleeding Outcomes, by DOAC Management Model (a Priori Analysis)
eFigure 8. Medication Persistence Among DOAC Users
eFigure 9. Association of Incident DOAC vs Warfarin Use and the Composite Endpoint in Subgroups
eFigure 10. Association of Incident DOAC vs Warfarin Use and the Composite Endpoint by Sensitivity Analysis
eReferences.

This supplementary material has been provided by the authors to give readers additional information about their work.

© 2023 Derington CG et al. *JAMA Network Open.*

eMethods.

Kaiser Permanente (KP) Health Plan and Delivery System

KP is an integrated healthcare plan and delivery system serving eight distinct regions of the United States (US). Each region operates according to its own local leadership, autonomy, and flexibility to establish and maintain services unique to the local population needs. Members are incentivized to seek inpatient and outpatient care through KP facilities to reduce medical costs and improve quality of care, although out-of-network care may be pursued for higher cost. Each region operates through a network of medical offices, outpatient surgery centers, laboratories, hospitals, pharmacies, and contracted facilities to care for its members.

This study was conducted in three of the eight regions: KP Northwest, KP Southern California, and KP Colorado. As of March 2022, the KP Northwest region serves >650,000 members, KP Southern California serves >4.6 million members, and KP Colorado serves >630,000 members.

Virtual Data Warehouse (VDW)

Each participating KP site employs a set of dataset standards and automated processes that allow programs written at one site to be run at other sites quickly and with minimal site-specific customization. The VDW records longitudinal data on patients' diagnoses captured with International Classification of Diseases (ICD)-9 and -10 codes; health services procedure data as captured with Healthcare Common Procedure Coding System, including Current Procedural Terminology (CPT) codes and Diagnosis-Related Group codes; pharmacy dispensing data that are captured with Generic Product Identifier codes and National Drug Codes; vitals, and laboratory measurements; patient demographics; geographic socioeconomic status as defined by the US Census; KP enrollment and membership data; and incidence and cause of death as captured by ICD-10 codes.

Warfarin and DOAC management model details

Pharmacists and nurses in the warfarin anticoagulation management services (AMS) have specialized training in the coordination and management of warfarin therapy. The pharmacists educate patients, order relevant laboratory tests including international normalized ratios, adjust warfarin doses, and establish peri-procedural plans for interruption of warfarin therapy in collaboration with a referring physician. In KPCO, interventions and international normalized ratio (INR) management occurs using the DAWN AC software, a centralized electronic database that interfaces with the EHR to interpret labs, formulate treatment plans, and document changes.[1] KPNW and KPSC utilize the EHR software (Epic Systems, Verona, WI) to manage anticoagulation therapy.

For DOAC UC group, the clinician who prescribed the DOAC managed all aspects of therapy as they would with any other prescription. The region's pharmacist-led AMS—which manage warfarin therapy—had no role in management of DOAC therapy but was available if consulted to provide peri-procedural DOAC therapy guidance.

In the UC + population management tool (PMT) DOAC model, Potential DOAC-related medication problems include non-adherence, potential drug-drug interactions, past due renal function labs, and dosing not matching renal function, age, and/or body weight, if applicable. After reviewing the weekly reports, the pharmacist contacted the prescribing clinician or patient, as appropriate, to address the identified problem.

In the AMS DOAC model, upon being enrolled in the service, patients were bonded to an individual pharmacist. Pharmacists in the AMS care for both patients using warfarin or DOAC therapy. Pharmacists evaluated each patient for DOAC appropriateness, dose-indication match, potential drug-drug interactions, needed laboratory measurements and dose adjustments. They also provided comprehensive education, including administration details, signs of bleeding or stroke, and drug storage, among other items. Thereafter, the patient was longitudinally assessed and contacted for adherence, new drug-drug interactions, laboratory monitoring, and patient education as needed. Renal function was assessed at least annually (every 6 months if the patient's calculated creatinine clearance was <60 mL/min). Drug interaction reports were reviewed daily, with critical drug interactions addressed immediately and noncritical drug interactions documented without specific follow up. Perioperative anticoagulation plans were developed and reviewed with patients as needed.

Indirect comparison with warfarin as a common comparator vs direct comparison of DOAC care models

Our primary research question was, "do patients initiating a DOAC require or benefit from specialized care management models (i.e., EHR-based tools or pharmacist management) beyond UC?" The most straightforward way to answer this question is by directly comparing outcomes of patients who received a DOAC but were managed under different care models. However, each DOAC care model is perfectly correlated with the KP region in which it was implemented. Therefore, the potential for biased treatment effect estimates caused by minor differential misclassification of outcomes (e.g., accuracy of diagnostic codes varying between regions) or unmeasured confounding due to population differences (e.g., unmeasured aspects of socioeconomic status varying between regions) exists, leading to potentially incorrect inferences. Fortunately, warfarin therapy is similarly managed across each KP region included in this analysis (i.e., each site uses a pharmacist-led anticoagulation clinic to manage warfarin therapy) and represents an ideal common comparator across all regions. KP also uses a national Anticoagulation Quality Improvement Work Group with representation from each KP region to track anticoagulation-related quality metrics, including warfarin time-in-therapeutic (TTR) range. Tracking these internal metrics has found minimal differences in TTR across regions, with all regions reporting TTR >70%. We, therefore, chose *a priori* to conduct three independent comparative effectiveness studies. Within each region, we compared patients who initiated a DOAC, who, by definition, received the DOAC care model implemented in that region, vs patients who initiated warfarin in that same region.

1. DOAC UC vs warfarin
2. DOAC UC + PMT vs warfarin
3. DOAC AMS vs warfarin

Our assumption with this approach was that by restricting the comparison to patients who live in the same region, we were less likely to have outcome misclassification which was differential to exposure or confounding by site level characteristics. By estimating unbiased treatment effects of each DOAC care model vs warfarin, then we could indirectly compare the DOAC care models.

This approach of using warfarin to conduct an indirect comparison of DOAC care models has known limitations. For example, indirect comparisons may suffer from low power and are less intuitive to understand. A direct comparison, whereby each DOAC care model is directly

© 2023 Derington CG et al. *JAMA Network Open.*

compared across sites, overcomes these limitations, but as stated above has its own limitation of potentially biased effect estimates due to unmeasurable confounding that exists between regions. However, the degree to which unmeasured confounding is an actual problem is unknown and there was disagreement within our research team regarding whether an indirect or direct comparison approach was the most appropriate approach to address our research question. Ultimately, our original submission to the *Journal* only included an indirect comparison. We received feedback from both peer-reviewers and the editor to include a direct comparison of the DOAC models as this approach is most intuitive.

In a *post hoc* analysis, we again leveraged warfarin therapy and the equivalent way it is managed across the participating KP regions. We empirically tested the likelihood that site level confounding may exist under the following logical assumptions.

1. We assume that no differences exist in clinical outcomes with warfarin across the 3 sites which are due to the management of warfarin.

2. If differences in warfarin outcomes exist, they are due to differences in patient characteristics which influence risk.

3. If the treatment effect estimates of warfarin in one region vs warfarin in another region (i.e., the hazard ratio [HR] and 95% confidence intervals [CI]) approach 1.0 after multivariable adjustment of all patient characteristics which are potential confounders, then we can conclude that there is a low likelihood of site-level confounding.

4. Conversely, if the treatment effect substantially departs for 1.0, then we can conclude there is a high likelihood of unmeasured confounding, either at the patient or site-level.

5. If we conclude that there is a low likelihood of site-level confounding in the warfarin analysis, then we can also conclude there is a low likelihood of site-level confounding in the DOAC analysis using the same analytic techniques.

We constructed propensity scores and IP weights within warfarin users. The education status variable was not balanced after applying the IP weights for the UC+PMT vs. UC comparison. Therefore, education was included in final analysis model. Weights were stabilized. All direct warfarin vs. warfarin comparisons demonstrated no significant differences in the net clinical benefit outcome as demonstrated in the table below.

	**Number of events (% per year)**			**IP-Weighted Hazard Ratio (95% CI)**	
**Outcome**	**Warfarin new users in KPNW (i.e., DOAC UC model) (N=2,885)**	**Warfarin new users in KPSC (i.e., DOAC UC + PMT model) (N=11,734)**	**Warfarin new users KPCO (i.e., DOAC AMS model) (N=2,850)**	**Usual Care + PMT vs Usual Care**	**AMS vs Usual Care**

© 2023 Derington CG et al. *JAMA Network Open.*

Net clinical benefit*	637 (9.07)	2897 (10.54)	534 (8.02)	0.92 (0.79,1.08)	0.95 (0.83,1.07)
*Composite of thromboembolic stroke, intracranial hemorrhage, gastrointestinal bleed, extracranial major bleed, and all cause death.<br>Number of events are unweighted, percent with events per year is weighted.<br>AMS: anticoagulation management service; CI: confidence interval; IP: inverse probability; PMT: population management tool.					

We, therefore, proceeded with conducting direct DOAC vs DOAC comparisons using the same analytic techniques. We did not compare the UC+PMT model directly against the AMS model because this comparison was outside the scope of our research question.

1. UC+PMT (KPSC model) vs. UC (KPNW model)

2. AMS (KPCO model) vs. UC (KPNW model)

For full transparency, we refer to the indirect comparison approach as an *a priori* analysis and the direct comparison approach as a *post-hoc* analysis.

Medication Adherence and Persistence
We excluded from the adherence analysis those who initiated warfarin at index because warfarin dosing can vary during treatment based on INR results without a new prescription being dispensed. Meaning, the pharmacy claims variable of “days supply dispensed” cannot be consistently relied upon. Direct oral anticoagulant (DOAC) users who switched to warfarin during the observation period (n = 1,519, 3.4% of all users) were excluded from the adherence analysis but included for the persistence analysis. We also restricted the persistence analysis to DOAC users at 12 months who were still alive and had KP membership. That is, patients were still included in the persistence analysis if they had an outcome of interest prior to 12 months.

We defined adherence to DOAC using the proportion of days covered (PDC). PDC was calculated as: $PDC = \frac{\text{Total number days supply of all DOAC prescriptions dispensed to the patient}}{\text{total number of days in the observation period}}$x100%.[2] The numerator was defined as the number of days’ supplied of all DOAC prescriptions for the patient, based on prescription fill dates and number of pills dispensed, in the total observation period. To calculate the observation period, we identified the start of the observation period as the day of first prescription fill, and the end of the observation as will be any of the following: thromboembolic stroke, intracranial hemorrhage, gastrointestinal bleed, extracranial major bleed, end of health plan enrollment, death, or one-year post-index date. We dichotomized the PDC outcome as <80% (ie, nonadherent) vs. ≥80% (ie, adherent). Calculations were estimated allowing stockpiling medications.

Medication persistence is defined as “the duration of time from the initiation and discontinuation of therapy.”[3] This can be identified in records by continued dispensing of the prescription. Discontinuation of therapy was defined as a gap of >50% of the previous prescription’s days’ supply.[4] For example, if a patient who received a 30-days’ supply on March 1st did not have another refill before April 16th, the patient was considered to have discontinued therapy. We identified patients who were persistent at 6 months (including the 180th day), then separately, patients who were persistent at 12 months (including the 365th day).

Calculation of CHA_2DS_2-VASc risk score and ATRIA bleed risk score

The CHA2DS_2-VASc risk score can be used to calculate a patient's risk for experiencing a stroke in the setting of non-valvular atrial fibrillation without anticoagulation.[5] We further categorized patients into low risk (score <2) vs. intermediate/high risk (score ≥2) based on their pre-index baseline characteristics.[6] The score is calculated as:

Criterion	Points
Age	<65: 0 points<br>65-74: 1 point<br>≥75: 2 points
Sex	Male: 0 points<br>Female: 1 point
History of congestive heart failure	No: 0 points<br>Yes: 1 point
History of hypertension	No: 0 points<br>Yes: 1 point
History of stroke, TIA, or systemic embolism	No: 0 points<br>Yes: 2 points
History of vascular disease (prior MI, peripheral artery disease, or aortic plaque)	No: 0 points<br>Yes: 1 point
History of diabetes	No: 0 points<br>Yes: 1 point
	Final score: sum from above

The ATRIA bleed risk score can be used to calculate a patient's risk for experiencing a minor or major bleeding event prior to taking warfarin therapy.[7] The score has been validated in real-world cohorts.[8] We further categorized patients into low risk (score <4) vs. intermediate/high risk (score ≥4) based on their pre-index baseline characteristics. The score is calculated as:

Criterion	Points
Presence of anemia<br>Men: hemoglobin <13 g/dL; women: hemoglobin <12 g/dL	No: 0 points<br>Yes: 3 points
Presence of severe renal disease<br>Glomerular filtration rate <30 mL/min or dependent on dialysis	No: 0 points<br>Yes: 3 points
Age ≥75 years	No: 0 points<br>Yes: 2 points
History of any prior hemorrhage diagnosis (example: GI bleed, intracranial hemorrhage)	No: 0 points<br>Yes: 1 point
History of hypertension	No: 0 points<br>Yes: 2 points
	Final score: sum from above

Covariate selection and generation of propensity score (PS) and inverse probability of treatment weights (IPTW)

To determine which of the candidate characteristics were included in our PS, we estimated two multivariable logistic regression models: 1) a treatment model (DOAC vs. warfarin, UC + PMT

© 2023 Derington CG et al. *JAMA Network Open.*

vs. UC, or AMS vs. UC); and 2) an outcome model (composite outcome occurred vs. the patient was censored during follow-up). The final PS models included all candidate characteristics except for those which were significantly associated with DOAC vs. warfarin use (p-value <0.05 in the treatment model) but not the outcome (p-value>0.15 in the outcome model).[9] Common support was assessed using histograms of the PS between groups.

We did not include in our final PS model those characteristics which were significantly associated with treatment (i.e., DOAC vs. warfarin in the *a priori* analysis, and UC+PMT vs. UC, then AMS vs. UC, in the direct analysis) (p-value <0.05 in the treatment model) but not the outcome (p-value>0.15 in the outcome model) as described by Brookhart et al.[9] First, using all covariates measured in Table 1, we conducted multivariable logistic regression to evaluate the association between the covariates and treatment ("treatment model"). Then, similarly using all covariates measured in Table 1, we conducted multivariable logistic regression to evaluate the association between the covariates and the primary composite outcome ("outcome model"). With the exception of age, sex, history of bleed, and year of index date, any covariate with a significant association in the treatment model (p<0.05) and an insignificant association in the outcome model (p>0.15) was removed from the final PS model.

After calculating a propensity score (PS) for each patient, we assigned each patient a weight based on the inverse of the PS. Thus, each patient is weighted by the reciprocal of the probability of receiving the treatment that he or she actually received. This weighting process creates a "pseudo-population" in which measured covariates are not confounded with treatment assignment, and therefore, we can estimate the average treatment effect between two treatment cohorts.[10] The inverse probability (IP) weights are calculated as:

$$IP\ weight = \frac{Z}{e} + \frac{1-Z}{1-e}$$

where *Z* indicates treatment status and *e* indicates the PS.

Thus, for a patient taking treatment (i.e., DOAC new users in the *a priori* analysis; receiving care in the UC+PMT or AMS models in the *post hoc* analysis) (Z = 1):

$$IP\ weight = \frac{1}{e} + \frac{1-1}{1-e} = \frac{\mathbf{1}}{\boldsymbol{e}}$$

And for a patient not taking treatment (i.e., warfarin new users in the *a priori* analysis; receiving care in the UC model in the *post hoc* analysis) (Z = 0):

$$IP\ weight = \frac{0}{e} + \frac{1-0}{1-e} = \frac{\mathbf{1}}{\mathbf{1}-\boldsymbol{e}}$$

For the *a priori* analysis, this process was repeated within each region. A final list of covariates in each region's PS model for the *a priori* analysis is shown in eTable 4. Weights were either stabilized or truncated at the 99th percentile, whichever resulted in greater balance (e.g., smaller absolute standardized mean difference). Weights were stabilized for the *post hoc* analyses.

Sensitivity Analyses
The first set of sensitivity analyses evaluated whether the results were sensitive to the chosen method of covariate adjustment, which was the use of inverse probability of treatment weighting with truncation of weights at the 1st and 99th percentiles. We repeated the analysis for the

© 2023 Derington CG et al. *JAMA Network Open.*

primary composite outcome and each component of the primary outcome using each method below. Results are presented alongside the unadjusted and primary results for comparison.

1) Minimal adjustment: Cox regression with adjustment for age and sex only.
2) Matching on the PS: Cox regression with a 1:1 PS matched population. Parameters included greedy algorithm with nearest-neighbor matching, caliper size equal to 0.2 of the standard deviation of the logit of the PS, without replacement.
3) Matching weights: Cox regression weighted according to “matching weights”. Matching weights provides an estimate of the average treatment effect and is an analog to PS matching, which only estimates the average treatment effect among the treated. Each patient’s matching weight can be calculated as:[11]

$$Matching\ weight = \frac{min(e_i, 1 - e_i)}{Z_i e_i + (1 - Z_i)(1 - e_i)}$$

where Z_i is the binary treatment indicator and e_i is the patient’s PS.

The second sensitivity analysis evaluated whether our results were sensitive to the construction of the cohort. To address this, we conducted the following series of analyses:

1) Complete case: Any patient missing any variable needed for the primary analysis was excluded from the cohort. Then, PS estimation and the primary analysis was repeated.
2) Exclusion of patients with a history of an event of interest: Any patient with an ICD-10 code in the 1-year pre-index period for any event in the composite outcome was excluded (see **eTable 2**). Then, PS estimation and the primary analysis was repeated.

The final set of sensitivity analysis, presented alongside the second set for simplicity, evaluated the timing of events and follow-up.

1) Early outcome events only: Inclusion/exclusion criteria, and primary analysis are the same, except that patients can also be censored at 90 days.
2) Late outcome events only: Patients who have any event of interest within the first 90 days (including the 90th day) are excluded from the cohort. Then, the PS is re-estimated, and analyses are repeated starting the observation time at day 91.
3) Restriction of events to the pre-COVID-19 pandemic period: patients were censored at the first occurrence of an outcome of interest, loss of KP membership, or December 31, 2019.

© 2023 Derington CG et al. *JAMA Network Open.*

eTable 1. STROBE Reporting Checklist for Cohort Studies

	Item No	Recommendation	Page No
**Title and abstract**	1	(*a*) Indicate the study's design with a commonly used term in the title or the abstract	3
		(*b*) Provide in the abstract an informative and balanced summary of what was done and what was found	3
**Introduction**			
Background/rationale	2	Explain the scientific background and rationale for the investigation being reported	5
Objectives	3	State specific objectives, including any prespecified hypotheses	5, eMethods
**Methods**			
Study design	4	Present key elements of study design early in the paper	5
Setting	5	Describe the setting, locations, and relevant dates, including periods of recruitment, exposure, follow-up, and data collection	5-9
Participants	6	(*a*) Give the eligibility criteria, and the sources and methods of selection of participants. Describe methods of follow-up	8
		(*b*) For matched studies, give matching criteria and number of exposed and unexposed	n/a
Variables	7	Clearly define all outcomes, exposures, predictors, potential confounders, and effect modifiers. Give diagnostic criteria, if applicable	9, 10 eTable 2, eTable 4
Data sources/ measurement	8*	For each variable of interest, give sources of data and details of methods of assessment (measurement). Describe comparability of assessment methods if there is more than one group	eTable 2
Bias	9	Describe any efforts to address potential sources of bias	eMethods
Study size	10	Explain how the study size was arrived at	12, eFigure 2
Quantitative variables	11	Explain how quantitative variables were handled in the analyses. If applicable, describe which groupings were chosen and why	10-11, eMethods, eTable 2
Statistical methods	12	(*a*) Describe all statistical methods, including those used to control for confounding	10-11
		(*b*) Describe any methods used to examine subgroups and interactions	12, eMethods
		(*c*) Explain how missing data were addressed	10, eTable 3
		(*d*) If applicable, explain how loss to follow-up was addressed	n/a
		(*e*) Describe any sensitivity analyses	eMethods
**Results**			
Participants	13*	(a) Report numbers of individuals at each stage of study—eg numbers potentially eligible, examined for eligibility, confirmed eligible, included in the study, completing follow-up, and analysed	12, eFigure 2
		(b) Give reasons for non-participation at each stage	12, eFigure 2
		(c) Consider use of a flow diagram	eFigure 2
Descriptive data	14*	(a) Give characteristics of study participants (eg demographic, clinical, social) and information on exposures and potential confounders	12 Table 1 eTable 5

© 2023 Derington CG et al. *JAMA Network Open.*

	Item No	Recommendation	Page No
		(b) Indicate number of participants with missing data for each variable of interest<br>(c) Summarise follow-up time (eg, average and total amount)	eTable 3<br><br>13
Outcome data	15*	Report numbers of outcome events or summary measures over time	Figure 1
Main results	16	(*a*) Give unadjusted estimates and, if applicable, confounder-adjusted estimates and their precision (eg, 95% confidence interval). Make clear which confounders were adjusted for and why they were included	Figure 1<br>eTable 8<br>eMethods
		(*b*) Report category boundaries when continuous variables were categorized	eTable 2
		(*c*) If relevant, consider translating estimates of relative risk into absolute risk for a meaningful time period	n/a
Other analyses	17	Report other analyses done—eg analyses of subgroups and interactions, and sensitivity analyses	14, eFigures 9-10
Discussion			
Key results	18	Summarise key results with reference to study objectives	15
Limitations	19	Discuss limitations of the study, taking into account sources of potential bias or imprecision. Discuss both direction and magnitude of any potential bias	8-9, 18
Interpretation	20	Give a cautious overall interpretation of results considering objectives, limitations, multiplicity of analyses, results from similar studies, and other relevant evidence	15-18
Generalisability	21	Discuss the generalisability (external validity) of the study results	18
Other information			
Funding	22	Give the source of funding and the role of the funders for the present study and, if applicable, for the original study on which the present article is based	20

© 2023 Derington CG et al. *JAMA Network Open.*

eTable 2. Variables Used to Define the Study Population, Baseline Covariates, Exposures, and Outcomes

**Variable**	**Definition**
Cohort creation	
Index date	The date of the first outpatient pharmacy dispensing for an oral anticoagulant prescription (warfarin or DOAC) between August 1, 2016, and December 31, 2019.
DOAC use	The first outpatient pharmacy dispensing for one of the following National Drug Codes:<br>a) Dabigatran (Pradaxa®): 00597013554, 00597013560, 00597014954, 00597014960, 00597010760, 00597010854, 00597010860, 00597035509, 00597036055, 00597036082, or 00597035556<br>b) Apixaban (Eliquis®): 00003089321, 00003089331, 00003089421, 00003089431, 00003089470, or 00003376474<br>c) Rivaroxaban (Xarelto®): 50458057890, 50458057910, 50458058030, 50458058010, 50458058090, 50458057810, 50458057830, 50458057710, 50458057760, 50458057718, 50458057930, 50458057990, 50458057989, or 50458058451<br>d) Edoxaban (Savaysa®): 65597020230, 65597020330, or 65597020390<br><br>An indicator flag was created for use of each specific DOAC agent.
Warfarin use	An outpatient pharmacy dispensing for one of the following National Drug Codes (this list includes generic warfarin, brand name Coumadin®, and brand name Jantoven®): 76282033301, 76282033310, 00056016870, 00056016970, 00056016975, 00056017070, 00056017270, 00056017370, 00056017470, 00056017670, 00056017690, 00056018870, 00093171901, 00555083102, 00555083202, 00555083205, 00555083302, 00555083402, 00555086902, 00555086905, 00555087402, 00555092502, 00555092602, 00832121200, 00832121300, 00832121400, 00832121800, 51672402701, 51672402703, 51672402801, 51672402803, 51672402901, 51672402903, 51672403001, 51672403101, 51672403203, 51672403301, 51672403401, 51672403501, 57237012201, 57237012301, 57237012701, 65162076311, 65162076411, 65162076511, 65162076610, 76282033201, 00056017290, 00056017375, 00056017475, 00056018875, 00555083105, 00555083305, 00555083502, 00590032435, 00832121100, 62584098401, 68084002701, 68382005201, 68382005601, 57237011999, 57237012199, 76282032910, 57237012299, 76282033010, 57237012399, 76282033110, 00093171201, 00093171210, 00832121101, 00832121110, 57237011901, 65162076110, 65162076111, 76282032701, 76282032710, 31722032701, 31722032710, 68382005210, 00179139070, 00093172001, 00832121950, 65162076910, 68382005901, 76282033501, 00832121900, 31722033501, 51672403503, 00056017075, 00093171301, 00093171310, 00832121210, 57237012001, 57237012099, 65162076210, 65162076211, 68382005301, 76282032801, 76282032810, 00056017090, 00406205310, 00832121201, 31722032801, 00056017001, 68382005310, 00056017675, 00093171401, 00093171410, 00832121301, 00832121310, 57237012101, 65162076310, 76282032901, 31722032901, 51672402907, 68382006401, 68382006410, 00093171501, 00832121410, 51672403003, 65162076410, 68382005401, 76282033001, 00406205410, 00832121401, 31722033001, 68382005410, 00179019170, 00093171601, 00093171610, 00832121500, 00832121510, 51672403103, 68382005501, 76282033101, 65162076510, 00056016875, 00555087405, 00056017275, 00093172101, 00093172110, 00832121600, 00832121610, 00832121689, 51672403201, 57237012401, 57237012499, 65162076611, 76282033210, 00832121601, 60429078910, 62584099401, 68382005610, 00179136670, 00093171801, 00832121710, 57237012501, 65162076711, 68382005701, 00056018975, 00832121700, 51672403303, 65162076710, 00056018970, 00093172301, 00832121850, 57237012601, 65162076810, 68382005801, 76282033401, 00832121801, 31722033401, 65162076811
Atrial fibrillation/flutter	An encounter with ICD-10 code I48.x (in any position) within 183 days prior or up to 7 days after cohort entry date (first dispensing date).

© 2023 Derington CG et al. *JAMA Network Open.*

Variable	Definition
Age	Age of patient calculated on the index date based on their date of birth. Categorized as: 18-64, 65-74, 75-84, and ≥85 for propensity score generation. Categorized as <65, 65-79, and ≥80 for subgroup analyses, based on DOAC dosing recommendations.
Non-continuous KP membership	Gap of greater than 45 days in KP medical benefit within 365 days prior to index date or 7 days after index date.
Pre-index death	Death date prior to the index date as determined by a death date present in Virtual Data Warehouse (VDW). Death data come from a variety of sources including the medical record, membership data and tumor registries, or state health departments or Geographically Enriched Member Sociodemographics (GEMS) a KP National datamart. Only death records marked as excellent confidence were included as they do not have any vital or encounter data present after the death date and are either supplied by the state health department or GEMS.
Baseline covariates	
Sex	Self-reported. Categorized as male or female
Race-ethnicity	Self-reported. Categorized as: Non-Hispanic Black, Non-Hispanic White, Hispanic, Asian, and Other for propensity score generation. Categorized as Non-Hispanic Black and all other race-ethnicities for subgroup analyses.
Smoking status	Self-reported. Categorized as: Never, Former, or Current.
Weight	The recorded weight prior and most proximal to index date. Categorized as <60 kg and ≥60 kg for subgroup analyses.
Body mass index	Discrete variable in VDW, derived from most recent weight and height entered into the medical record prior to the index date. Categorized as: <18.5, 18.5 to <25, 25 to <30, and ≥30, reported in kg/m^2
Area-level (US Census tract) education status	Reported based on zip code cross-referenced with the zip code/education status from the American Community Survey. Categorized as low (>20% of area-level residents did not graduate high school) or high.
Area-level (US Census tract) median income	Reported based on zip code cross-referenced with the zip code/median income from the American Community Survey. Categorized as low (<$50,000) or high (≥$50,000).
Systolic blood pressure	The systolic blood pressure (mm Hg) value within 365 days prior and most proximal to index date. Inpatient and emergency department measurements were excluded. If more than 1 measurement on the same day, the average was used. Categorized as low (<140) or high (≥140).
Diastolic blood pressure	The diastolic blood pressure (mm Hg) value within 365 days prior and most proximal to index date. Inpatient and emergency department measurements were excluded. If more than 1 measurement on the same day, the average was used. Categorized as low (<90) or high (≥90).
Total cholesterol	The total cholesterol (mg/dL) value within 365 days prior and most proximal to index date. Inpatient and emergency department measurements were excluded.
Low-density lipoprotein cholesterol	The LDL-C (mg/dL) value within 365 days prior and most proximal to index date. Inpatient and emergency department measurements were excluded.

© 2023 Derington CG et al. *JAMA Network Open.*

Variable	Definition
High-density lipoprotein cholesterol	The HDL-C (mg/dL) value within 365 days prior and most proximal to index date. Inpatient and emergency department measurements were excluded.
Serum glucose	The serum glucose (mg/dL) value within 365 days prior and most proximal to index date. Inpatient and emergency department measurements were excluded.
Hemoglobin A1c	The hemoglobin A1c (%) value within 365 days prior and most proximal to index date, from any setting. Categorized as <5.7%, 5.7 to <6.5%, and ≥6.5%.
Creatinine clearance	Calculated using the serum creatinine (mg/dL) value prior and most proximal to the index date, using the Cockroft-Gault equation using the patient's actual body weight recorded in the electronic health record. Inpatient and emergency department measurements of serum creatinine were excluded. Reported as mL/min. Categorized as <30, 30 to <45, 45 to <90 and ≥90 mL/min for propensity score generation. Categorized as <30, 30 to <50, and ≥50 for subgroup analyses, based on DOAC dosing recommendations.
ALT	The ALT (mg/dL) value within 365 days prior and most proximal to index date. Inpatient and emergency department measurements were excluded.
AST	The AST (mg/dL) value within 365 days prior and most proximal to index date. Inpatient and emergency department measurements were excluded.
Type of atrial fibrillation	First encounter of any type in the 183-day pre-index period with an AF ICD-10 diagnosis code of one of the following:<br>a) I48.0 = Paroxysmal<br>b) I48.1x = Persistent<br>c) I48.2x = Chronic<br>d) I48.3 or I48.4 = Flutter<br>e) I48.9x = Unspecified
Heart Failure	Encounter of any type in the 365-day pre-index period with an ICD-10 code of one of the following: I11.0, I13.0, I13.2, I42.0, or I50. If multiple encounters present, the code from the most recent encounter prior to index date was used.
Hypertension	Encounter of any type in the 183-day pre-index period with an ICD-10 code of one of the following: I10 through I16. If multiple encounters present, the code from the most recent encounter prior to index date was used.
Diabetes	Encounter of any type in the 183-day pre-index period with an ICD-10 code of one of the following: E10 or E11. If multiple encounters present, the code from the most recent encounter prior to index date was used.
History of transient ischemic attack	Encounter of any type in the 183-day pre-index period with an ICD-10 code of G45. If multiple encounters present, the code from the most recent encounter prior to index date was used.
History of myocardial infarction	Encounter of any type in the 183-day pre-index period with an ICD-10 code of one of the following: I21 through I23. If multiple encounters present, the code from the most recent encounter prior to index date was used.
History of peripheral artery disease	Encounter of any type in the 183-day pre-index period with an ICD-10 code of one of the following: I70.2-I70.9, I71, or I73.9. If multiple encounters present, the code from the most recent encounter prior to index date was used.
History of aortic plaque	Encounter of any type in the 183-day pre-index period with an ICD-10 code of I70.0. If multiple encounters present, the code from the most recent encounter prior to index date was used.

© 2023 Derington CG et al. *JAMA Network Open.*

Variable	Definition
History of vascular disease	Meeting criteria for history of myocardial infarction, history of peripheral artery disease, or history of aortic plaque, as defined above. Used for propensity score generation and for calculating the CHADS2VASc Score.
End-stage renal disease	Defined as patient with kidney transplant in the 365-day pre-index period defined as CPT codes: 00868, 50300, 50320, 50323, 50325, 50327, 50328, 50329, 50340, 50360, 50365, 50370, 50380, 50547 or ICD10 procedure codes: 0TY00Z0, 0TY00Z1, 0TY00Z2, 0TY10Z0, 0TY10Z1, 0TY10Z2 or patient is dialysis dependent as defined by CPT codes 49420, 49421, 90945, 36145, 36800, 36832, 36833, 90935, 93990 or ICD 10 procedure codes 3E1.M39Z, 05H.Y33Z, 06H.Y33Z, 031.30ZD, 031.40ZD, 031.509V, 031.50AV, 031.50JV, 031.50KV, 031.50ZD, 031.50ZV, 031.609V, 031.60AV, 031.60JV, 031.60KV, 031.60ZD, 031.60ZV, 031.709V, 031.70AV, 031.70JV, 031.70KV, 031.70ZD, 031.70ZV, 031.809V, 031.80AV, 031.80JV, 031.80KV, 031.80ZD, 031.80ZV, 031.90ZF, 031.A0ZF, 031.B0ZF, 031.C0ZF, 5A1.D70Z, 5A1.D80Z, 5A1.D90Z or ICD10 diagnosis codes Z49.02, T85.71XA, Z49.31, Z49.01. Used to calculate CHADS2VASc Score.
Moderate/severe renal disease	Encounter of any type in the 183-day pre-index period with an ICD-10 code of I12, I13, N00, N01, N02, N03, N04, N05, N07, N11, N14, N17, N18, N19, or Q61. If multiple encounters present, the code from the most recent encounter prior to index date was used. Used to calculate CHADS2VASc Score.
Intracranial hemorrhage	Definition derived from the Sentinel Initiative.[12] A binary variable indicating the occurrence of one ICD-10-CM diagnosis code from an inpatient encounter in the primary position of one of the following: I60.00, I60.01, I60.02, I60.10, I60.11, I60.12, I60.2, I60.30, I60.31, I60.32, I60.4, I60.50, I60.51, I60.52, I60.6, I60.7, I60.8, I60.9, I61.0, I61.1, I61.2, I61.3, I61.4, I61.5, I61.6, I61.8, I61.9, I62.00, I62.01, I62.02, I62.03, I62.1, I62.9, S06.340A, S06.341A, S06.342A, S06.343A, S06.344A, S06.345A, S06.346A, S06.347A, S06.348A, S06.349A, S06.350A, S06.351A, S06.352A, S06.353A, S06.354A, S06.355A, S06.356A, S06.357A, S06.358A, S06.359A, S06.360A, S06.361A, S06.362A, S06.363A, S06.364A, S06.365A, S06.366A, S06.367A, S06.368A, S06.369A, S06.4X0A, S06.4X1A, S06.4X2A, S06.4X3A, S06.4X4A, S06.4X5A, S06.4X6A, S06.4X7A, S06.4X8A, S06.4X9A, S06.5X0A, S06.5X1A, S06.5X2A, S06.5X3A, S06.5X4A, S06.5X5A, S06.5X6A, S06.5X7A, S06.5X8A, S06.5X9A, S06.6X0A, S06.6X1A, S06.6X2A, S06.6X3A, S06.6X4A, S06.6X5A, S06.6X6A, S06.6X7A, S06.6X8A, or S06.6X9A.
Gastrointestinal bleed	Definition derived from the Sentinel Initiative.[12] A binary variable indicating the occurrence of a GI bleed according to one of the following:<br>a) One ICD-10-CM diagnosis code from an inpatient encounter in the primary position of one of the following: I85.01, I85.11, K22.6, K22.8, K25.0, K25.2, K25.4, K25.6, K26.0, K26.2, K26.4, K26.6, K27.0, K27.2, K27.4, K27.6, K28.0, K28.2, K28.4, K28.6, K29.01, K29.21, K29.31, K29.41, K29.51, K29.61, K29.71, K29.81, K29.91, K31.811, K55.21, K56.699, K57.01, K57.11, K57.13, K57.21, K57.31, K57.33, K57.41, K57.51, K57.53, K57.81, K57.91, K57.93, K62.5, K64.0, K64.1, K64.2, K64.3, K64.4, K64.8, K66.1, K92.0, K92.1, K92.2, or R58.<br>b) One ICD-10-CM diagnosis code from an inpatient encounter in the any position of any of the codes listed in (a) above, AND: one ICD-10-CM diagnosis code from an inpatient encounter on the same day in the primary position of one of the following: K29.00, K29.20, K29.30, K29.40, K29.50, K29.60, K29.70, K29.80, K29.90, K57.00, K57.10, K57.12, K57.20, K57.30, K57.32, K57.40, K57.50, K57.52, K57.80, K57.90, K57.92, K64.0, K64.1, K64.2, K64.3, K64.4, K64.5, K64.8, or K64.9.
Extracranial major bleed	Definition derived from the Sentinel Initiative.[12] A binary variable indicating the occurrence of an extracranial major bleed according to one of the following:<br>a) One ICD-10-CM diagnosis code from an inpatient encounter in the primary position of one of the following: I31.2, I85.01, I85.11, K22.6, K22.8, K25.0, K25.2, K25.4, K25.6, K26.0, K26.2, K26.4, K26.6, K27.0, K27.2, K27.4, K27.6, K28.0, K28.2, K28.4, K28.6, K29.01, K29.21, K29.31, K29.41, K29.51, K29.61, K29.71, K29.81, K29.91, K31.811, K55.21, K56.699, K57.01, K57.11, K57.13, K57.21, K57.31, K57.33, K57.41, K57.51, K57.53, K57.81, K57.91, K57.93, K62.5, K64.0, K64.1, K64.2, K64.3, K64.4, K64.8, K66.1, K92.0, K92.1, K92.2, M25.00, M25.011, M25.012, M25.019, M25.021, M25.022, M25.029, M25.031, M25.032, M25.039, M25.041, M25.042, M25.049, M25.051, M25.052, M25.059, M25.061, M25.062, M25.069, M25.071, M25.072, M25.073, M25.074, M25.075, M25.076,

© 2023 Derington CG et al. *JAMA Network Open.*

Variable	Definition
	M25.08, N28.0, N89.8, N92.0, N92.1, R04.0, R04.1, R58, or 531.6, AND: no ICD-10-CM or CPT code indicating trauma during an encounter on the same day. Please refer to Sentinel Initiative website for full trauma ICD-10 and CPT codes.<br>b) One ICD-10-CM diagnosis code from an inpatient encounter in the primary position of one of the following: D50.0, D62, D64.9, K29.00, K29.20m K29.30, K29.40, K29.50, K29.60, K29.70, K29.80, , K29.90, K57.00, K57.10, K57.12, K57.20, K57.30, K57.32, K57.40, K57.50, K57.52, K57.80, K57.90, K57.92, K64.0, K64.1, K64.2, K64.3, K64.4, K64.5, K64.8, K64.9, or R79.1. AND: one code in the secondary or unspecified position listed in a) above, AND no ICD-10-CM or CPT code indicating trauma during an encounter on the same day. Please refer to Sentinel Initiative website for full trauma ICD-10 and CPT codes.
History of bleed	Meeting criteria for history of intracranial hemorrhage, gastrointestinal bleeding, or extracranial major bleeding, as defined above. Used for propensity score generation and for calculating the CHA2DS2VASc Score.
Alcohol abuse	Encounter of any type in the 183-day pre-index period with an ICD-10 code of one of the following: E22.4, E52.9A F10, G31.2, G62.1, G72.1, I42.6, K29.2, K70, K86.0, L27.8A, O35.4 T51, Z71.4, Z72.1. If multiple encounters present, the code from the most recent encounter prior to index date was used.
History of anemia	The presence of anemia in the 365-day pre-index to 90-day after window according to the hemoglobin value (g/dL) prior and most proximal to index date. Inpatient and emergency department measurements were excluded. A hemoglobin <12 g/dL in women and <13 g/dL in men indicated presence of anemia. Used to calculate the ATRIA bleed risk score.
Hospitalization	Count variable of the number of hospitalizations in the 365-day pre-index period for any cause. Further categorized into <1 and ≥1.
Emergency department visits	Count variable of the number of emergency department visits in the 365-day pre-index period for any cause. Further categorized into <1 and ≥1.
Ambulatory care visit	Count variable of the number of anticoagulation-related ambulatory care visits in the 365-day pre-index period. Further categorized into <1 and ≥1.
CHA_2DS_2-VASc score	Continuous variable, calculated as described in eMethods. Categorized into low risk (score <2) vs. intermediate/high risk (score ≥2) for subgroup analyses.
ATRIA bleed score	Continuous variable, calculated as described in eMethods. Categorized into low risk (score <4) vs. intermediate/high risk (score ≥4) for subgroup analyses.
Outcomes[12,13]	
Thromboembolic stroke	Definition derived from the Sentinel Initiative.[12] A binary variable indicating the occurrence one ICD-10-CM diagnosis code from an inpatient encounter in the primary position of one of the following: I63.22, I63.139, I63.239, I63.019, I63.119, I63.219, I63.59, I63.20, I63.30, I63.40, I63.50, I67.89, I63.00, I63.011, I63.012, I63.013, I63.02, I63.031, I63.032, I63.033, I63.039, I63.09, I63.10, I63.111, I63.112, I63.113, I63.12, I63.131, I63.132, I63.133, I63.19, I63.211, I63.212, I63.213, I63.231, I63.232, I63.233, I63.29, I63.311, I63.312, I63.313, I63.319, I63.321, I63.322, I63.323, I63.329, I63.331, I63.332, I63.333, I63.339, I63.341, I63.342, I63.343, I63.349, I63.39, I63.411, I63.412, I63.413, I63.419, I63.421, I63.422, I63.423, I63.429, I63.431, I63.432, I63.433, I63.439, I63.441, I63.442, I63.443, I63.449, I63.49, I63.511, I63.512, I63.513, I63.519, I63.521, I63.522, I63.523, I63.529, I63.531, I63.532, I63.533, I63.539, I63.541, I63.542, I63.543, I63.549, I63.6, I63.8, or I63.9.
Intracranial hemorrhage	Definition derived from the Sentinel Initiative.[12] A binary variable indicating the occurrence of one ICD-10-CM diagnosis code from an inpatient encounter in the primary position of one of the following: I60.00, I60.01, I60.02, I60.10, I60.11, I60.12, I60.2, I60.30, I60.31, I60.32, I60.4, I60.50, I60.51, I60.52, I60.6, I60.7, I60.8, I60.9, I61.0, I61.1, I61.2, I61.3, I61.4, I61.5, I61.6, I61.8, I61.9, I62.00, I62.01, I62.02, I62.03, I62.1,

© 2023 Derington CG et al. *JAMA Network Open.*

Variable	Definition
	I62.9, S06.340A, S06.341A, S06.342A, S06.343A, S06.344A, S06.345A, S06.346A, S06.347A, S06.348A, S06.349A, S06.350A, S06.351A, S06.352A, S06.353A, S06.354A, S06.355A, S06.356A, S06.357A, S06.358A, S06.359A, S06.360A, S06.361A, S06.362A, S06.363A, S06.364A, S06.365A, S06.366A, S06.367A, S06.368A, S06.369A, S06.4X0A, S06.4X1A, S06.4X2A, S06.4X3A, S06.4X4A, S06.4X5A, S06.4X6A, S06.4X7A, S06.4X8A, S06.4X9A, S06.5X0A, S06.5X1A, S06.5X2A, S06.5X3A, S06.5X4A, S06.5X5A, S06.5X6A, S06.5X7A, S06.5X8A, S06.5X9A, S06.6X0A, S06.6X1A, S06.6X2A, S06.6X3A, S06.6X4A, S06.6X5A, S06.6X6A, S06.6X7A, S06.6X8A, or S06.6X9A.
Gastrointestinal bleed	Definition derived from the Sentinel Initiative.[12] A binary variable indicating the occurrence of a GI bleed according to one of the following:<br>a) One ICD-10-CM diagnosis code from an inpatient encounter in the primary position of one of the following: I85.01, I85.11, K22.6, K22.8, K25.0, K25.2, K25.4, K25.6, K26.0, K26.2, K26.4, K26.6, K27.0, K27.2, K27.4, K27.6, K28.0, K28.2, K28.4, K28.6, K29.01, K29.21, K29.31, K29.41, K29.51, K29.61, K29.71, K29.81, K29.91, K31.811, K55.21, K56.699, K57.01, K57.11, K57.13, K57.21, K57.31, K57.33, K57.41, K57.51, K57.53, K57.81, K57.91, K57.93, K62.5, K64.0, K64.1, K64.2, K64.3, K64.4, K64.8, K66.1, K92.0, K92.1, K92.2, or R58.<br>b) One ICD-10-CM diagnosis code from an inpatient encounter in the any position of any of the codes listed in (a) above, AND: one ICD-10-CM diagnosis code from an inpatient encounter on the same day in the primary position of one of the following: K29.00, K29.20, K29.30, K29.40, K29.50, K29.60, K29.70, K29.80, K29.90, K57.00, K57.10, K57.12, K57.20, K57.30, K57.32, K57.40, K57.50, K57.52, K57.80, K57.90, K57.92, K64.0, K64.1, K64.2, K64.3, K64.4, K64.5, K64.8, or K64.9.
Extracranial major bleed	Definition derived from the Sentinel Initiative.[12] A binary variable indicating the occurrence of an extracranial major bleed according to one of the following:<br>a) One ICD-10-CM diagnosis code from an inpatient encounter in the primary position of one of the following: I31.2, I85.01, I85.11, K22.6, K22.8, K25.0, K25.2, K25.4, K25.6, K26.0, K26.2, K26.4, K26.6, K27.0, K27.2, K27.4, K27.6, K28.0, K28.2, K28.4, K28.6, K29.01, K29.21, K29.31, K29.41, K29.51, K29.61, K29.71, K29.81, K29.91, K31.811, K55.21, K56.699, K57.01, K57.11, K57.13, K57.21, K57.31, K57.33, K57.41, K57.51, K57.53, K57.81, K57.91, K57.93, K62.5, K64.0, K64.1, K64.2, K64.3, K64.4, K64.8, K66.1, K92.0, K92.1, K92.2, M25.00, M25.011, M25.012, M25.019, M25.021, M25.022, M25.029, M25.031, M25.032, M25.039, M25.041, M25.042, M25.049, M25.051, M25.052, M25.059, M25.061, M25.062, M25.069, M25.071, M25.072, M25.073, M25.074, M25.075, M25.076, M25.08, N28.0, N89.8, N92.0, N92.1, R04.0, R04.1, R58, or 531.6, AND: no ICD-10-CM or CPT code indicating trauma during an encounter on the same day. Please refer to Sentinel Initiative website for full trauma ICD-10 and CPT codes.<br>b) One ICD-10-CM diagnosis code from an inpatient encounter in the primary position of one of the following: D50.0, D62, D64.9, K29.00, K29.20m K29.30, K29.40, K29.50, K29.60, K29.70, K29.80, , K29.90, K57.00, K57.10, K57.12, K57.20, K57.30, K57.32, K57.40, K57.50, K57.52, K57.80, K57.90, K57.92, K64.0, K64.1, K64.2, K64.3, K64.4, K64.5, K64.8, K64.9, or R79.1. AND: one code in the secondary or unspecified position listed in a) above, AND no ICD-10-CM or CPT code indicating trauma during an encounter on the same day. Please refer to Sentinel Initiative website for full trauma ICD-10 and CPT codes.
Death	Death date following the index date and prior to December 31, 2020, as determined by a death date present in VDW. Only death records marked as excellent confidence were included.
Composite outcome	Binary variable indicating the occurrence of thromboembolic stroke, intracranial hemorrhage, gastrointestinal bleed, extracranial major bleed, or death, as defined above, in the follow-up period starting 7 days post-index.

© 2023 Derington CG et al. *JAMA Network Open.*

eTable 3. Missingness of Characteristics Used to Generate the Propensity Score, by KP Region

Patient characteristics	Overall<br>N = 44,746	Usual care<br>N = 6,182	Usual care + PMT<br>N = 33,625	AMS<br>N = 4,939
Demographics				
Age	0 (0.0)	0 (0.0)	0 (0.0)	0 (0.0)
Sex	0 (0.0)	0 (0.0)	0 (0.0)	0 (0.0)
Race-ethnicity	860 (1.9)	36 (0.6)	722 (2.2)	102 (2.1)
Tobacco use	679 (1.5)	53 (0.9)	586 (1.7)	40 (0.8)
Area-level education	499 (1.1)	1 (0.02)	497 (1.5)	1 (0.02)
Area-level income	531 (1.2)	1 (0.02)	506 (1.5)	24 (0.5)
Vitals and laboratory measurements				
Body mass index, kg/m^2	478 (1.1)	155 (2.5)	197 (0.6)	126 (2.6)
Systolic BP, mm Hg	256 (0.6)	66 (1.1)	112 (0.3)	78 (1.6)
Diastolic BP, mm Hg	256 (0.6)	66 (1.1)	112 (0.3)	78 (1.6)
Creatinine Clearance	1411 (3.2)	315 (5.1)	966 (2.9)	130 (2.6)
Medical History				
Type of AF	0 (0.0)	0 (0.0)	0 (0.0)	0 (0.0)
Alcohol abuse	0 (0.0)	0 (0.0)	0 (0.0)	0 (0.0)
Anemia (component of ATRIA bleed score)	7248 (16.2)	1240 (20.1)	5190 (15.4)	818 (16.6)
Any baseline outcome bleed	0 (0.0)	0 (0.0)	0 (0.0)	0 (0.0)
Congestive heart failure	0 (0.0)	0 (0.0)	0 (0.0)	0 (0.0)
Diabetes	0 (0.0)	0 (0.0)	0 (0.0)	0 (0.0)
Hypertension	0 (0.0)	0 (0.0)	0 (0.0)	0 (0.0)
Moderate-severe liver disease	0 (0.0)	0 (0.0)	0 (0.0)	0 (0.0)
Stroke from CHA_2DS_2-VASc score*	0 (0.0)	0 (0.0)	0 (0.0)	0 (0.0)
Vascular disease from CHA_2DS_2-VASc score†	0 (0.0)	0 (0.0)	0 (0.0)	0 (0.0)
Healthcare visits during pre-index period				
Ambulatory care visits	0 (0.0)	0 (0.0)	0 (0.0)	0 (0.0)
Emergency department visits	0 (0.0)	0 (0.0)	0 (0.0)	0 (0.0)
Hospitalizations	0 (0.0)	0 (0.0)	0 (0.0)	0 (0.0)

Numbers in table are frequency (column %) with missing data.
*Defined as ischemic stroke, systemic embolism, or transient ischemic attack
†Defined as myocardial infarction, peripheral artery disease, or aortic plaque
AF: atrial fibrillation; ALT: alanine transaminase; AMS: anticoagulation management service; AST: aspartate aminotransferase; BMI: body mass index; BP: blood pressure; GI: gastrointestinal; HDL: high-density lipoprotein cholesterol; KP: Kaiser Permanente; LDL: low-density lipoprotein cholesterol; PMT: population management tool

© 2023 Derington CG et al. *JAMA Network Open.*

eTable 4. Characteristics Included in the Final Propensity Score Model (DOAC vs Warfarin) for the a Priori Analysis

Characteristics	Usual care	Usual care + PMT	AMS
Year of index date	X	X	X
Age	X	X	X
Sex	X	X	X
Race-ethnicity	X	X	
Tobacco use	X	X	X
Low area-level education	X	X	X
Low area-level income	X	X	X
BMI, kg/m^2	X	X	X
High systolic BP, mm Hg	X	X	X
High diastolic BP, mm Hg	X	X	X
Creatinine Clearance, mL/min	X	X	X
Type of AF	X	X	
Comorbidities			
Alcohol abuse	X	X	
Anemia (component of ATRIA bleed score)	X	X	X
Any baseline outcome bleed	X	X	X
Congestive heart failure	X	X	X
Diabetes	X	X	X
Hypertension	X	X	
Moderate-severe liver disease	X	X	X
Stroke from CHA_2DS_2-VASc score*	X	X	X
Vascular disease from CHA_2DS_2-VASc score†	X	X	X
Healthcare utilization			
Any ED visit baseline		X	X
Any inpatient visit baseline	X	X	X
Any ambulatory care visit baseline	X		

An “X” indicates that the variable was included in the generation of the propensity score for that KP region. See eTable 1 for categorization of each variable.
Age, sex, and year of index date were forced into each region’s model.
*Defined as ischemic stroke, systemic embolism, or transient ischemic attack
†Defined as myocardial infarction, peripheral artery disease, or aortic plaque
AF: atrial fibrillation; AMS: anticoagulation management service; BMI: body mass index; BP: blood pressure; ED: emergency department; GI: gastrointestinal; ICH: intracranial hemorrhage; PMT: population management tool

© 2023 Derington CG et al. *JAMA Network Open.*

eTable 5. Complete Set of Characteristics of DOAC and Warfarin Users Across Three DOAC Management Models, Before Weighting and Multiple Imputation

	Usual care		Usual care + PMT		AMS	
	**DOAC**	**Warfarin**	**DOAC**	**Warfarin**	**DOAC**	**Warfarin**
**No. of patients***	N=3297	N=2885	N=21891	N=11734	N=2089	N=2850
Dabigatran	3061 (93.0)	-	20044 (91.6)	-	1749 (83.8)	-
Apixaban	149 (4.5)	-	1379 (6.3)	-	251 (12.0)	-
Rivaroxaban	83 (2.5)	-	457 (2.1)	-	86 (4.1)	-
**Year of index date**						
2016	225 (6.8)	567 (19.7)	1728 (7.9)	3883 (33.1)	190 (9.1)	543 (19.1)
2017	720 (21.8)	1074 (37.2)	5474 (25.0)	4441 (37.9)	524 (25.1)	998 (35.0)
2018	1004 (30.5)	776 (26.9)	6933 (31.7)	2062 (17.6)	606 (29.0)	748 (26.3)
2019	1348 (40.9)	468 (16.2)	7756 (35.4)	1348 (11.5)	769 (36.8)	561 (19.7)
**Demographics**						
Age, years	71.3 (10.6)	73.9 (10.1)	72.2 (11.1)	74.2 (10.9)	72.5 (10.5)	74.6 (10.3)
18-64	727 (22.1)	450 (15.6)	4708 (21.5)	2056 (17.5)	395 (18.9)	430 (15.1)
65-74	1269 (38.5)	1004 (34.8)	7579 (34.6)	3544 (30.2)	766 (36.7)	900 (31.6)
75-84	989 (30.0)	1004 (34.8)	6863 (31.4)	4137 (35.3)	678 (32.5)	1036 (36.4)
≥85	312 (9.5)	427 (14.8)	2741 (12.5)	1997 (17.0)	250 (12.0)	484 (17.0)
Sex						
Male	1836 (55.7)	1562 (54.1)	12431 (56.8)	6602 (56.3)	1172 (56.1)	1509 (53.0)
Female	1461 (44.3)	1322 (45.8)	9459 (43.2)	5132 (43.7)	917 (43.9)	1341 (47.1)
Race-ethnicity						
Asian	48 (1.5)	53 (1.8)	1687 (7.7)	1010 (8.6)	25 (1.2)	30 (1.1)
Hispanic	61 (1.9)	60 (2.1)	4243 (19.4)	2548 (21.7)	136 (6.5)	229 (8.0)
Non-Hispanic Black	42 (1.3)	43 (1.5)	1471 (6.7)	993 (8.5)	56 (2.7)	82 (2.9)
Non-Hispanic White	3027 (91.8)	2604 (90.3)	13548 (61.9)	6732 (57.4)	1776 (85.0)	2366 (83.0)
Unknown/ Missing	23 (0.7)	13 (0.5)	526 (2.4)	196 (1.7)	54 (2.6)	48 (1.7)
Other†	96 (2.9)	112 (3.9)	416 (1.9)	255 (2.2)	42 (2.0)	95 (3.3)
Lives in a census tract where:						
>20% of residents have less than a high school degree	386 (11.7)	330 (11.4)	6002 (27.4)	3659 (31.2)	206 (9.9)	367 (12.9)
Annual household income <$50,000 USD	1097 (33.3)	968 (33.6)	3448 (15.8)	2110 (18.0)	416 (19.9)	679 (23.8)
Tobacco use						
Current	131 (4.0)	132 (4.6)	722 (3.3)	339 (2.9)	92 (4.4)	136 (4.8)
Former	1593 (48.3)	1452 (50.3)	9549 (43.6)	5637 (48.0)	989 (47.3)	1421 (49.9)
Never	1538 (46.7)	1283 (44.5)	11211 (51.2)	5581 (47.6)	984 (47.1)	1277 (44.8)
Unknown/Missing	35 (1.1)	18 (0.6)	409 (1.9)	177 (1.5)	24 (1.2)	16 (0.6)
**Physiologic Variables**						

	Usual care		Usual care + PMT		AMS	
Weight, kg	91.6 (24.1)	92.3 (26.5)	87.0 (24.0)	84.8 (24.2)	85.4 (22.2)	85.6 (23.5)
>60 kg	2970 (90.1)	2606 (90.3)	19440 (88.8)	10135 (86.4)	1824 (87.3)	2452 (86.0)
BMI, kg/m2	31.1 ± 7.3	31.7 ± 8.4	29.8 ± 7	29.5 ± 7.2	28.9 ± 6.5	29.6 ± 7.1
<18.5	26 (0.8)	39 (1.4)	325 (1.5)	212 (1.8)	33 (1.6)	49 (1.7)
18.5 to <25	591 (17.9)	527 (18.3)	5142 (23.5)	3082 (26.3)	528 (25.3)	701 (24.6)
25 to <30	1018 (30.9)	806 (27.9)	7240 (33.1)	3763 (32.1)	729 (34.9)	919 (32.3)
≥30	1567 (47.5)	1453 (50.4)	9054 (41.4)	4610 (39.3)	734 (35.1)	1120 (39.3)
Systolic BP, mm Hg	127.1 ± 17.5	126.7 ± 18.1	127.8 ± 16.3	126.9 ± 16.6	123.2 ± 16.8	124.4 ± 17
High (≥140)	674 (20.4)	601 (20.8)	4417 (20.2)	2262 (19.3)	252 (12.1)	394 (13.8)
Diastolic BP, mm Hg	71.9 ± 12	70.4 ± 12	71.6 ± 11.9	68.8 ± 11.6	71.9 ± 10.7	71.5 ± 11.1
High (≥90)	225 (6.8)	179 (6.2)	1351 (6.2)	427 (3.6)	98 (4.7)	152 (5.3)
Serum glucose, mg/dL	118.3 ± 45.3	121.5 ± 46.7	131.2 ± 56	134.7 ± 59.2	109.8 ± 37.2	113.8 ± 43.1
Hemoglobin A1c, %	6.3 ± 1.2	6.5 ± 1.2	6.3 ± 1.2	6.5 ± 1.2	6.2 ± 1.1	6.4 ± 1.3
<5.7	636 (19.3)	424 (14.7)	4677 (21.4)	1850 (15.8)	435 (20.8)	447 (15.7)
5.7 to <6.5	934 (28.3)	853 (29.6)	7264 (33.2)	3721 (31.7)	601 (28.8)	754 (26.5)
≥6.5	586 (17.8)	713 (24.7)	4876 (22.3)	3513 (29.9)	298 (14.3)	577 (20.3)
HDL cholesterol, mg/dL	50.4 ± 16.5	48.5 ± 15.8	49.8 ± 15.2	47.6 ± 14.6	51.2 ± 16.3	49.7 ± 16.2
LDL cholesterol, mg/dL	91.2 ± 37.5	87.9 ± 37.3	88.1 ± 33.6	82.5 ± 32.5	85.5 ± 33.3	80.8 ± 33
Total cholesterol, mg/dL	166.1 ± 43.1	159.7 ± 43.5	160 ± 41.5	153 ± 40.8	163.6 ± 40.9	157.9 ± 41.9
Creatinine Clearance, mL/min	91.7 ± 42.1	81.4 ± 43.7	84.4 ± 39.4	70.1 ± 41.2	80.5 ± 33.3	75.7 ± 37.4
<30	45 (1.4)	195 (6.8)	549 (2.5)	1509 (12.9)	37 (1.8)	180 (6.3)
30 to <45	203 (6.2)	356 (12.3)	1914 (8.7)	1734 (14.8)	185 (8.9)	343 (12.0)
45 to <90	1478 (44.8)	1259 (43.6)	10986 (50.2)	5336 (45.5)	1136 (54.4)	1465 (51.4)
≥90	1382 (41.9)	949 (32.9)	7734 (35.3)	2897 (24.7)	673 (32.2)	790 (27.7)
AST, mg/dL	33.7 ± 23.8	36.2 ± 95.5	30.6 ± 40.5	32.3 ± 51.3	34.3 ± 76.3	38.4 ± 180.5
ALT, mg/dL	33.1 ± 30	35.7 ± 102.3	26.4 ± 30.6	26.7 ± 45.6	33.4 ± 60.9	36.2 ± 114.5
**Stroke and Bleed Risk Scores**						
CHA_2DS_2-VASc	3 [2-5]	4 [3-5]	4[2-5]	4 [3-5]	3 [2-5]	4 [3-5]
ATRIA stroke risk	6 [5-8]	7 [5-9]	7 [5-8]	8 [6-9]	7 [5-8]	7 [5-8]
ATRIA bleed risk	2 [1-3]	3 [1-5]	2 [1-3]	3 [1-6]	2 [1-3]	3 [1-4]
**Medical Conditions**						
Type of AF/Flutter						
Atrial flutter	30 (0.9)	8 (0.3)	107 (0.5)	28 (0.2)	47 (2.3)	30 (1.1)
Paroxysmal	1377 (41.8)	1066 (37.0)	9843 (45.0)	4605 (39.2)	1025 (49.1)	1178 (41.3)
Persistent	115 (3.5)	107 (3.7)	673 (3.1)	341 (2.9)	120 (5.7)	103 (3.6)
Chronic	286 (8.7)	256 (8.9)	944 (4.3)	914 (7.8)	119 (5.7)	177 (6.2)

© 2023 Derington CG et al. *JAMA Network Open.*

	Usual care		Usual care + PMT		AMS	
Unspecified or unknown	1489 (45.2)	1448 (50.2)	10324 (47.16)	5846 (49.8)	778 (37.2)	1362 (47.8)
Alcohol abuse	136 (4.1)	97 (3.4)	976 (4.5)	486 (4.1)	81 (3.9)	83 (2.9)
History of thromboembolic stroke	179 (5.4)	155 (5.4)	1055 (4.8)	442 (3.8)	153 (7.3)	173 (6.1)
History of GI bleed	6 (0.2)	16 (0.6)	87 (0.4)	91 (0.8)	19 (0.9)	42 (1.5)
History of Intracranial Bleed	8 (0.2)	7 (0.2)	98 (0.5)	61 (0.5)	16 (0.8)	27 (1.0)
History of Extracranial Major Bleed	8 (0.2)	19 (0.7)	97 (0.4)	101 (0.9)	19 (0.9)	44 (1.5)
Diabetes mellitus	872 (26.5)	1053 (36.5)	7354 (33.6)	5113 (43.6)	506 (24.2)	844 (29.6)
Heart failure	845 (25.6)	977 (33.9)	5559 (25.4)	4900 (41.8)	568 (27.2)	997 (35.0)
Hypertension	2279 (69.1)	2113 (73.2)	17040 (77.8)	10011 (85.3)	1393 (66.7)	2114 (74.2)
History of myocardial infarction	258 (7.8)	245 (8.5)	1140 (5.2)	901 (7.7)	134 (6.4)	151 (5.3)
Peripheral artery disease	322 (9.8)	368 (12.8)	1637 (7.5)	1395 (11.9)	199 (9.5)	310 (10.9)
Moderate-severe liver disease	29 (0.9)	44 (1.5)	126 (0.6)	142 (1.2)	14 (0.7)	32 (1.1)
Moderate-severe renal disease	838 (25.4)	1086 (37.6)	6305 (28.8)	5746 (49.0)	656 (31.4)	1272 (44.6)
**Healthcare encounters during pre-index period**						
Ambulatory care visits	9 [5-15]	10 [5-18]	10 [5-17]	13 [7-21]	7 [4-11]	7 [4-11]
Emergency department visits	1 [0-2]	1 [0-2]	1 [0-2]	1 [0-2]	0 [0-1]	0 [0-1]
Hospitalizations	0 [0-1]	1 [0-1]	0 [0-1]	1 [0-1]	0 [0-1]	1 [0-1]

Numbers represent n (%), mean ± SD, or median [IQR] unless otherwise specified.
The index date was the date of first direct oral anticoagulant pharmacy fill; all values were collected in the one year prior to the index date.
*Does not add up to 100% in each region because some patients were on multiple DOACs (UC n=4; UC + PMT n=9; AMS n=3).
† Includes patients who self-report as any non-Hispanic ethnicity and at least one of the following races: American Indian / Alaska Native, Native Hawaiian or Other Pacific Islander, Other (not specified), or more than one race.
AF: atrial fibrillation; ALT: alanine transaminase; AMS: anticoagulation management services; AST: aspartate aminotransferase; BMI: body mass index; BP: blood pressure; DOAC: direct oral anticoagulant; GI: gastrointestinal; HDL: high-density lipoprotein cholesterol; LDL: low-density lipoprotein cholesterol; PMT: population management tool; USD: United States Dollars

© 2023 Derington CG et al. *JAMA Network Open.*

eTable 6. Association of DOAC Management Models With Individual Bleeding Outcomes

Outcome	Number of events (% per year)			IP-Weighted Hazard Ratio (95% CI)	
	Usual Care (N=3,297)	Usual Care + PMT (N=21,891)	AMS (N=2,089)	Usual Care + PMT vs Usual Care	AMS vs Usual Care
**Net clinical benefit***	6 (0.09)	134 (0.32)	5 (0.11)	2.77 (0.41,18.82)	1.22 (0.05,32.19)
**Thromboembolic stroke**	45 (0.67)	296 (0.71)	47 (1.06)	0.97 (0.52,1.81)	1.31 (0.83,2.05)
**Major bleeding****	45 (0.67)	296 (0.71)	48 (1.08)	0.97 (0.52,1.81)	1.37 (0.88,2.15)
**All-cause death**	6 (0.09)	134 (0.32)	5 (0.11)	2.77 (0.41,18.82)	1.22 (0.05,32.19)

*Composite of thromboembolic stroke, intracranial hemorrhage, gastrointestinal bleed, extracranial major bleed, and all cause death.
**Composite of intracranial hemorrhage, gastrointestinal bleeding, and extracranial major bleeding.
Number of events are unweighted, percent with events per year is weighted.
AMS: anticoagulation management service; CI: confidence interval; IP: inverse probability; PMT: population management tool.

© 2023 Derington CG et al. *JAMA Network Open.*

eTable 7. One-Year Medication Adherence Rates Among DOAC Users

Outcome	Usual care N = 3,062	Usual care + PMT N = 20,790	AMS N = 1,906
**Medication adherence**			
Average, PDC (SD)	0.81 (0.30)	0.79 (0.30)	0.81 (0.31)
PDC ≥80% n (%)	2,193 (71.6%)	14,256 (68.6%)	1,380 (72.4%)

See eMethods for descriptions of how adherence metrics are calculated. DOAC sample was restricted to patients with one year of follow-up.
AMS: anticoagulation management services; DOAC: direct oral anticoagulant; PDC: proportion of days' covered; PMT: population management tool

© 2023 Derington CG et al. *JAMA Network Open.*

eTable 8. Hazard Ratios for the Primary Outcomes, by Covariate Adjustment Strategy (a Priori Analysis)

	**Usual care**	**Usual care + PMT**	**AMS**
**Outcome and adjustment strategy**	**HR for DOAC vs. warfarin (95% CI)**	**HR for DOAC vs. warfarin (95% CI)**	**HR for DOAC vs. warfarin (95% CI)**
**Composite endpoint***			
Crude	0.54 (0.47,0.61)	0.52 (0.49,0.54)	0.58 (0.50,0.68)
Minimally-adjusted**	0.62 (0.55,0.71)	0.57 (0.54,0.60)	0.66 (0.56,0.77)
Propensity score matching	0.85 (0.73,1.00)	0.85 (0.79.0.82)	0.83 (0.69,1.01)
Propensity score strata	0.87 (0.76,1.00)	0.83 (0.78,0.88)	0.84 (0.71,0.98)
Matching weight adjusted	0.90 (0.78,1.04)	0.89 (0.83,0.94)	0.85 (0.72,1.00)
Primary analysis: IPTW (truncate to 99%ile)	0.91 (0.79,1.05)	0.85 (0.79,0.90)	0.84 (0.72,0.99)
**Thromboembolic stroke**			
Crude	0.61 (0.40,0.95)	0.92 (0.77,1.10)	0.69 (0.45,1.06)
Minimally-adjusted**	0.71 (0.46,1.11)	0.97 (0.81,1.16)	0.74 (0.48,1.14)
Propensity score matching	0.89 (0.50,1.56)	1.18 (0.91,1.52)	0.84 (0.51,1.39)
Propensity score strata	0.95 (0.58,1.54)	1.12 (0.81,1.37)	0.86 (0.55,1.35)
Matching weight adjusted	0.95 (0.58,1.55)	1.16 (0.94,1.43)	0.89 (0.56,1.40)
Primary analysis: IPTW (truncate to 99%ile)	0.97 (0.95,1.59)	1.15 (0.92,1.43)	0.84 (0.54,1.33)
**Intracranial hemorrhage**			
Crude	0.18 (0.07,0.43)	0.39 (0.31,0.48)	0.14 (0.06,0.35)
Minimally-adjusted**	0.20 (0.08,0.48)	0.41 (0.33,0.51)	0.16 (0.06,0.41)
Propensity score matching	0.23 (0.07,0.74)	0.53 (0.38,0.74)	0.15 (0.06,0.39)
Propensity score strata	0.22 (0.09,0.56)	0.50 (0.39, 0.65)	0.16 (0.06,0.40)
Matching weight adjusted	0.22 (0.08,0.61)	0.52 (0.40,0.68)	0.17 (0.07,0.43)
Primary analysis: IPTW (truncate to 99%ile)	0.22 (0.08,0.56)	0.50 (0.38,0.65)	0.19 (0.07,0.50)
**Gastrointestinal bleed**			
Crude	0.71 (0.48,1.05)	0.57 (0.49,0.67)	0.89 (0.62,1.29)
Minimally-adjusted**	0.79 (0.53,1.17)	0.60 (0.51,0.71)	0.99 (0.68,1.43)
Propensity score matching	0.96 (0.57,1.59)	0.96 (0.76,1.20)	1.16 (0.72,1.86)
Propensity score strata	1.06 (0.69,1.64)	0.94 (0.78,1.14)	1.20 (0.82,1.78)
Matching weight adjusted	1.00 (0.65,1.54)	1.02 (0.85,1.24)	1.18 (0.79,1.75)
Primary analysis: IPTW (truncate to 99%ile)	1.21 (0.78,1.89)	0.88 (0.72,1.08)	1.19 (0.81,1.76)
**Extracranial major bleed**			
Crude	0.64 (0.44,0.94)	0.54 (0.46,0.63)	0.80 (0.56,1.14)
Minimally-adjusted**	0.71 (0.48,1.04)	0.56 (0.48,0.66)	0.89 (0.62,1.27)

© 2023 Derington CG et al. *JAMA Network Open.*

	Usual care	Usual care + PMT	AMS
Propensity score matching	0.85 (0.52,1.40)	0.88 (0.70,1.10)	1.02 (0.65,1.60)
Propensity score strata	0.93 (0.61,1.42)	0.85 (1.70,1.02)	1.04 (0.72,1.52)
Matching weight adjusted	0.89 (0.59,1.36)	0.92 (0.76,1.12)	1.04 (0.71,1.53)
Primary analysis: IPTW (truncate to 99%ile)	1.09 (0.71,1.68)	0.79 (0.65,0.97)	1.03 (0.71,1.51)
**Death**			
Crude	0.54 (0.47,0.62)	0.49 (0.46,0.52)	0.55 (0.46,0.66)
Minimally-adjusted**	0.63 (0.55,0.73)	0.54 (0.51,0.58)	0.63 (0.52,0.76)
Propensity score matching	0.92 (0.78,1.10)	0.85 (0.78,0.92)	0.85 (0.67,1.07)
Propensity score strata	0.93 (0.80,1.08)	0.82 (0.77,0.88)	0.84 (0.69,1.02)
Matching weight adjusted	0.98 (0.84,1.14)	0.89 (0.83,0.95)	0.85 (0.70,1.04)
Primary analysis: IPTW (truncate to 99%ile)	0.96 (0.82,1.11)	0.85 (0.79,0.92)	0.85 (0.70,1.03)

* Composite endpoint of thromboembolic stroke, intracranial hemorrhage, gastrointestinal bleed, extracranial major bleed, or death.

** Age and sex only.

AMS: anticoagulation management services; CI: confidence interval; DOAC: direct oral anticoagulant; HR: hazard ratio; IPTW: inverse propensity treatment weighting; PMT: population management tool

© 2023 Derington CG et al. *JAMA Network Open.*

eFigure 1. Study Design Schema

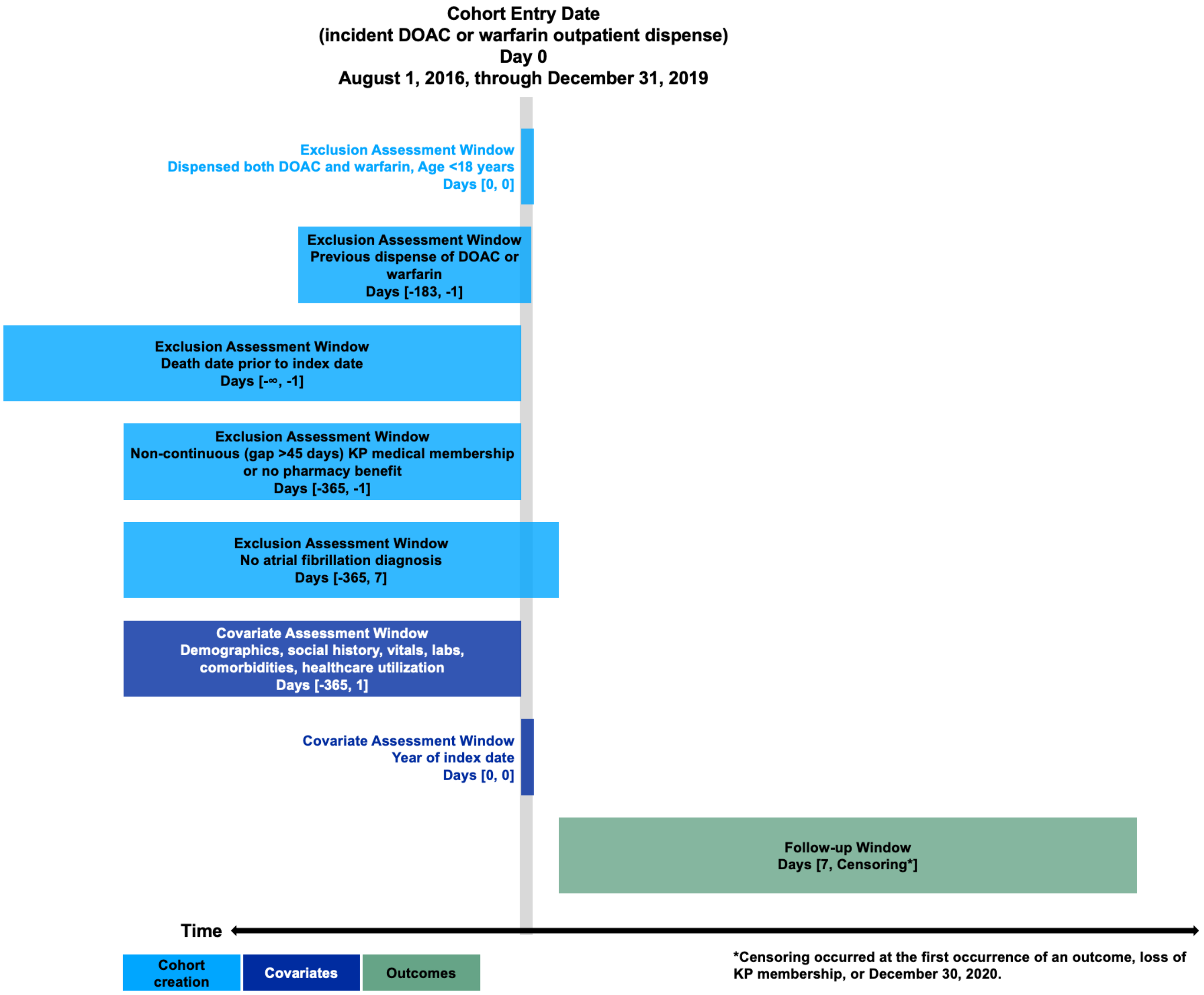

Abbreviations: DOAC: direct oral anticoagulant; KP: Kaiser Permanente

© 2023 Derington CG et al. *JAMA Network Open.*

eFigure 2. Flowchart for Patient Inclusion in the Current Study

Panel A: Usual care

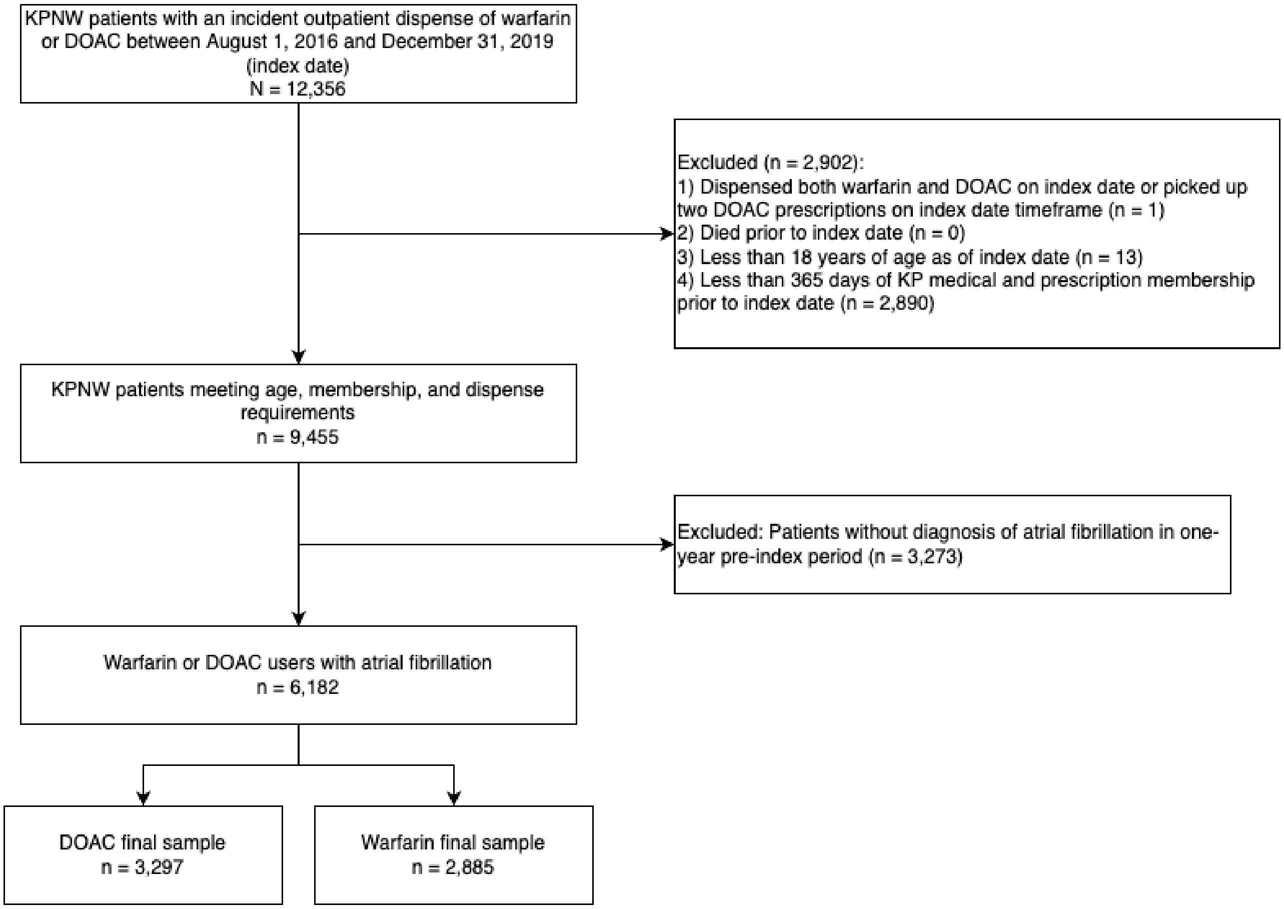

© 2023 Derington CG et al. *JAMA Network Open.*

Panel B: Usual care + PMT

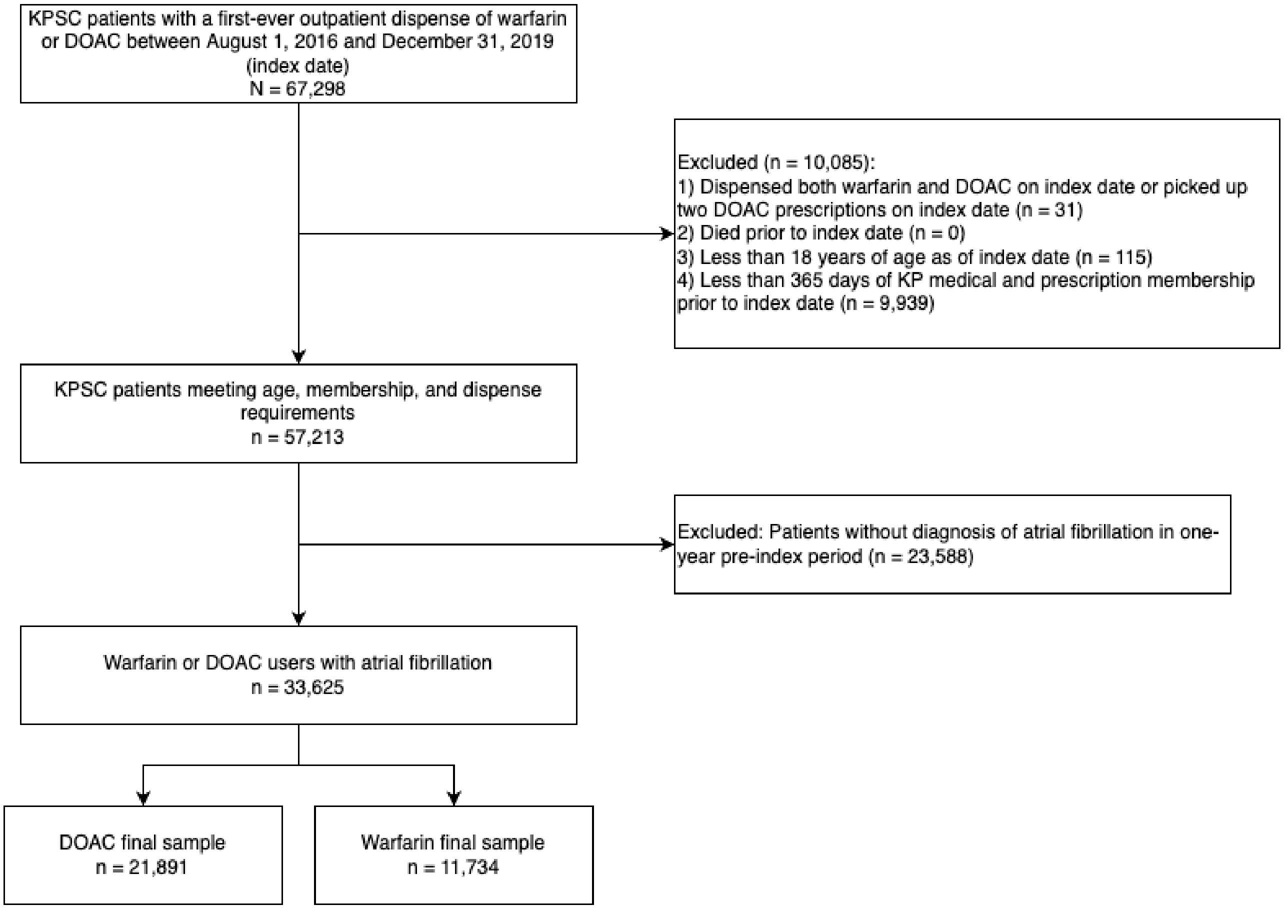

© 2023 Derington CG et al. *JAMA Network Open.*

Panel C: AMS

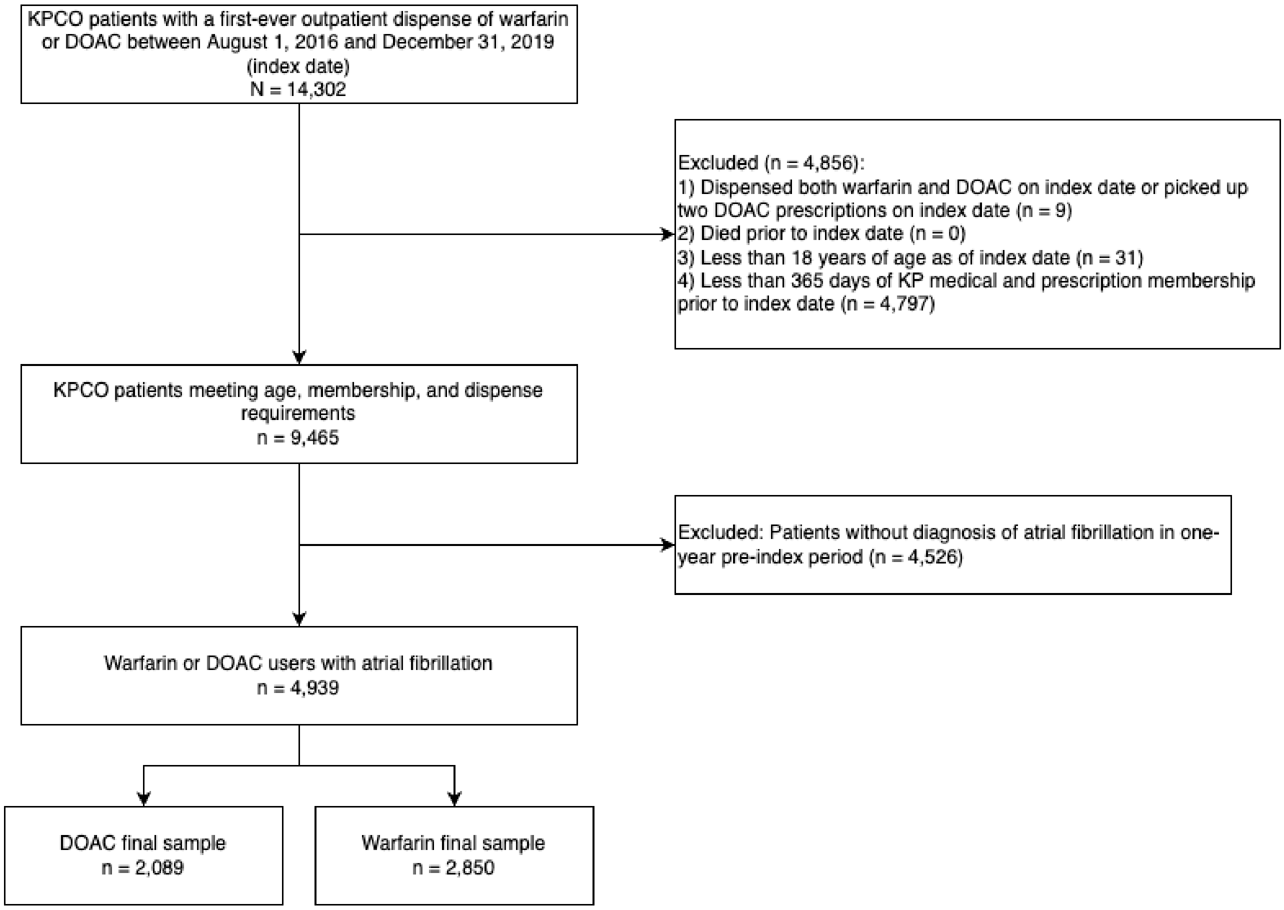

Abbreviations: AMS: anticoagulation management services; DOAC: direct oral anticoagulant; KP: Kaiser Permanente; PMT: population management tool

© 2023 Derington CG et al. *JAMA Network Open.*

eFigure 3. Propensity Score Histograms for Being a DOAC vs Warfarin User (a Priori Analysis)

Panel A: Usual care

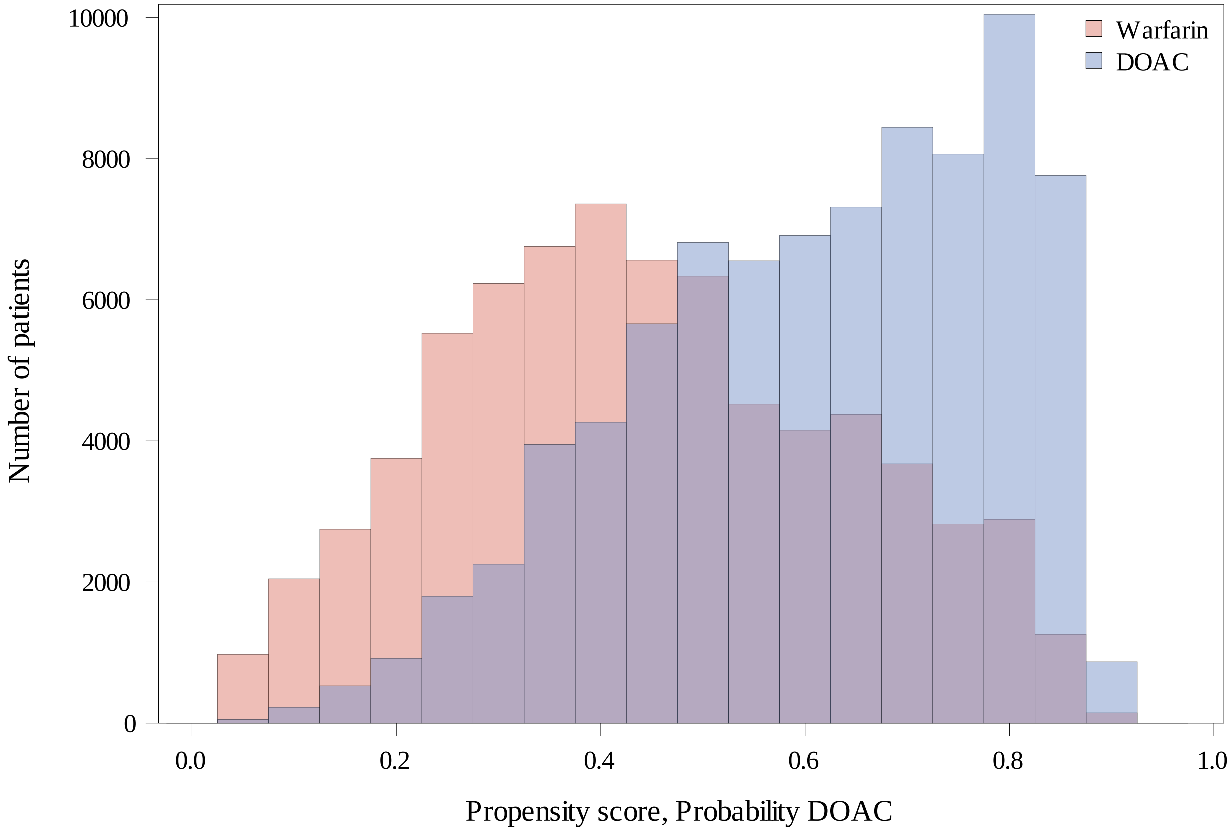

© 2023 Derington CG et al. *JAMA Network Open.*

Panel B: Usual care + PMT

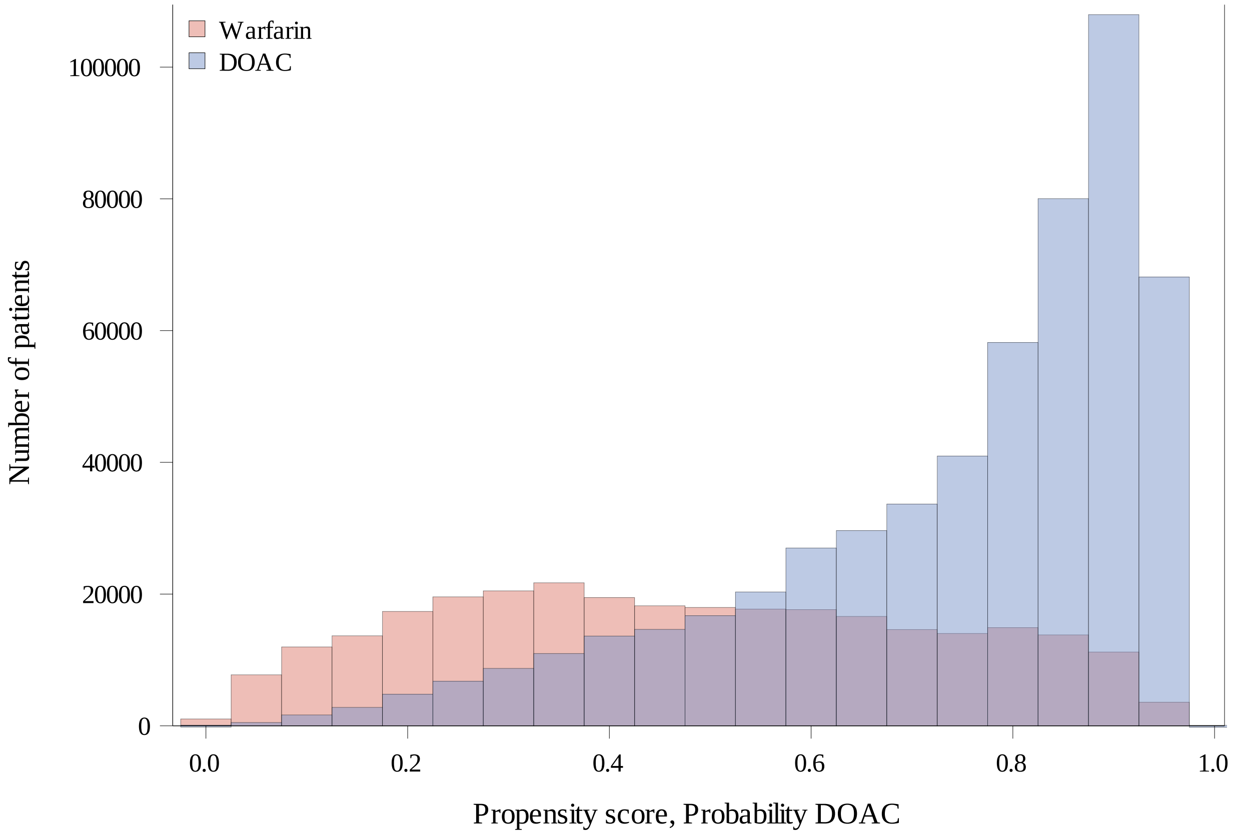

© 2023 Derington CG et al. *JAMA Network Open.*

Panel C: AMS

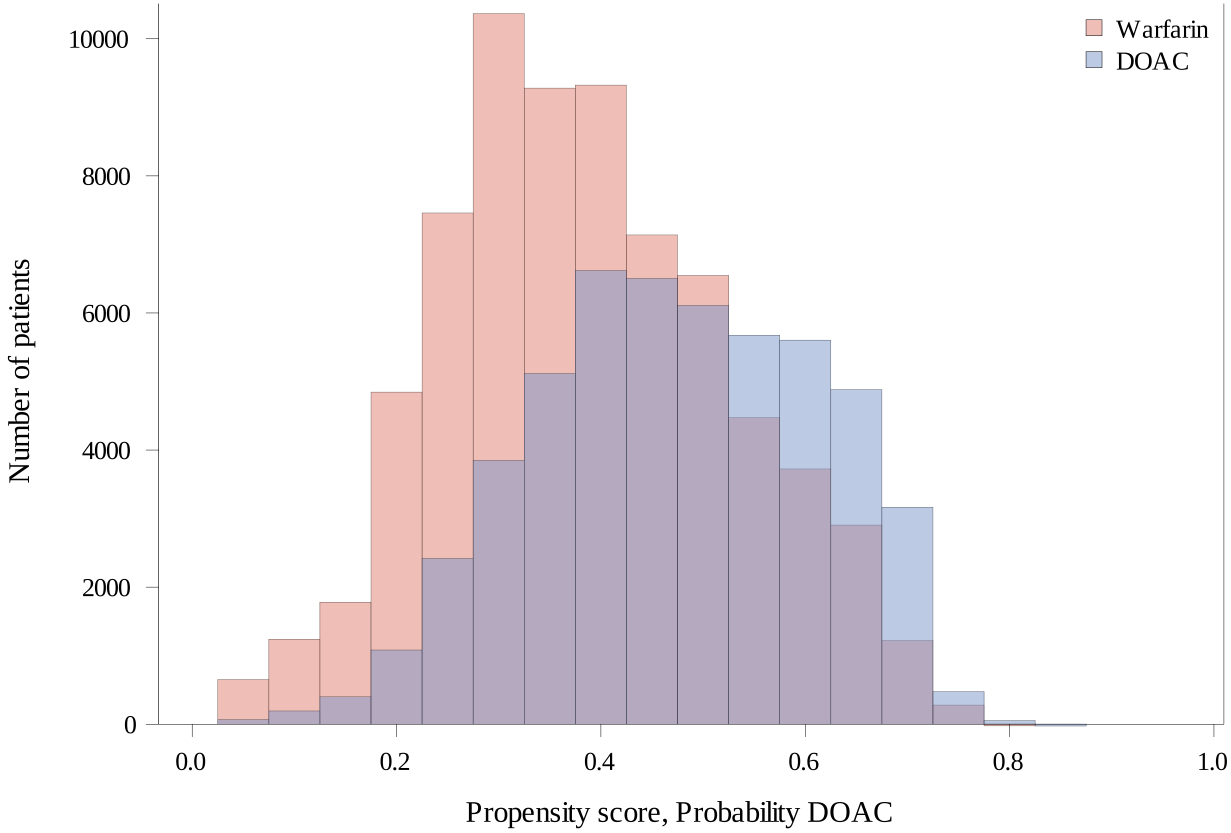

Abbreviations: AMS: anticoagulation management services; DOAC: direct oral anticoagulant; PMT: population management tool

© 2023 Derington CG et al. *JAMA Network Open.*

eFigure 4. Balance of Patient Characteristics Before and After Inverse Probability of Treatment Weighting (a Priori Analysis)

Panel A: Usual care model

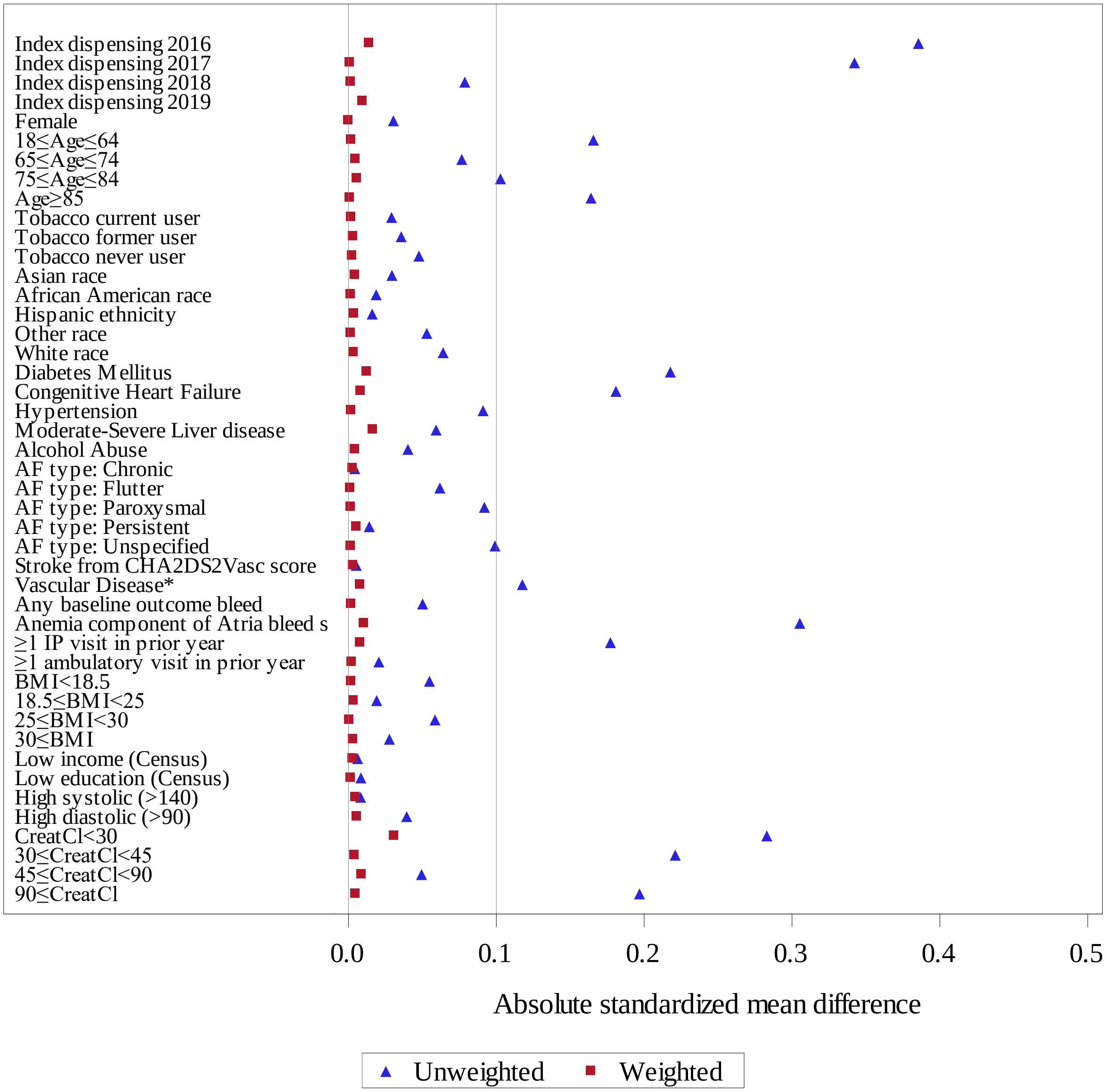

*Vascular Disease: MI/Peripheral Artery Disease/Aortic plaque

© 2023 Derington CG et al. *JAMA Network Open.*

Panel B: UC + PMT DOAC care model

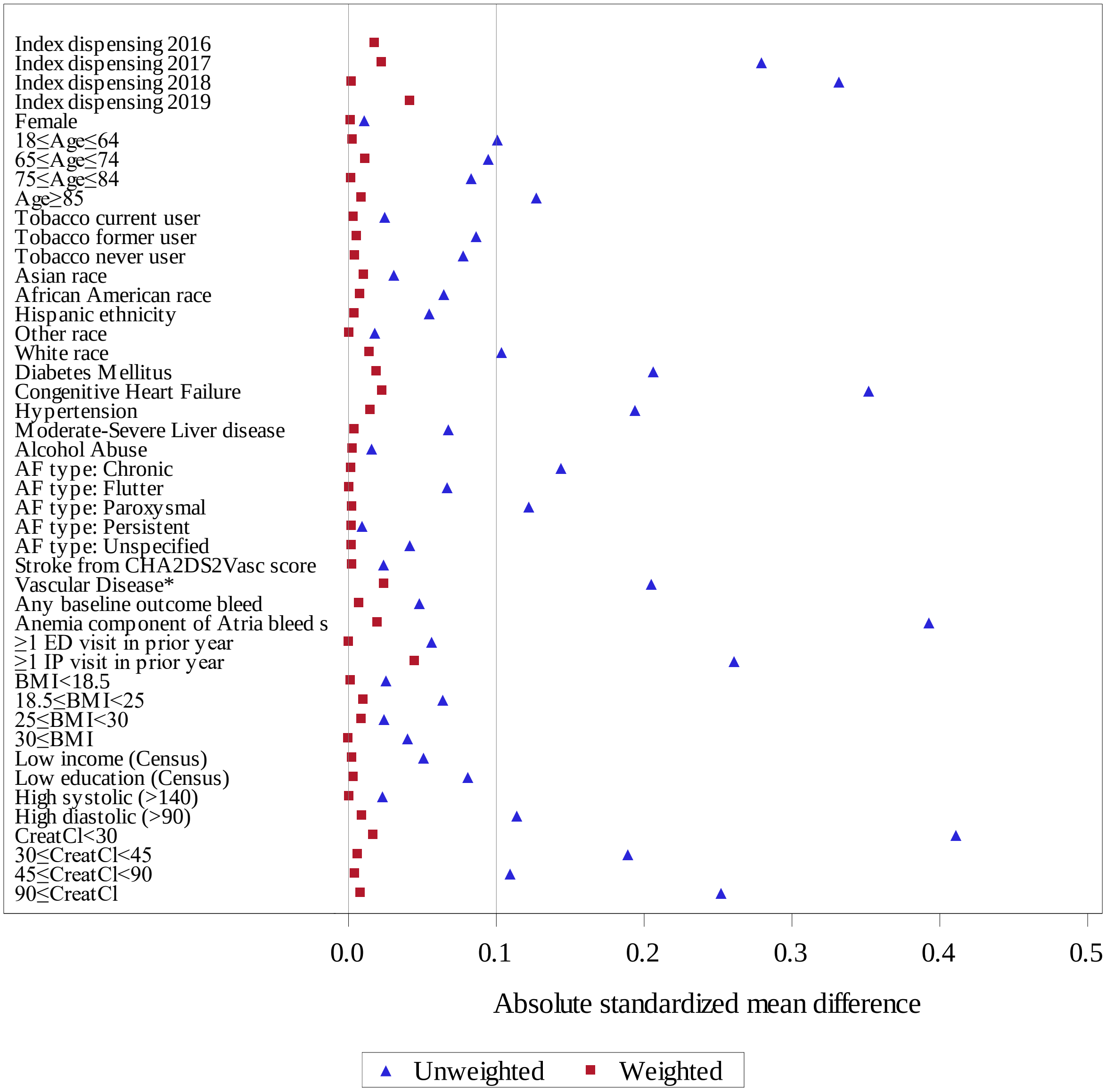

*Vascular Disease: MI/Peripheral Artery Disease/Aortic plaque

© 2023 Derington CG et al. *JAMA Network Open.*

Panel C: AMS care model

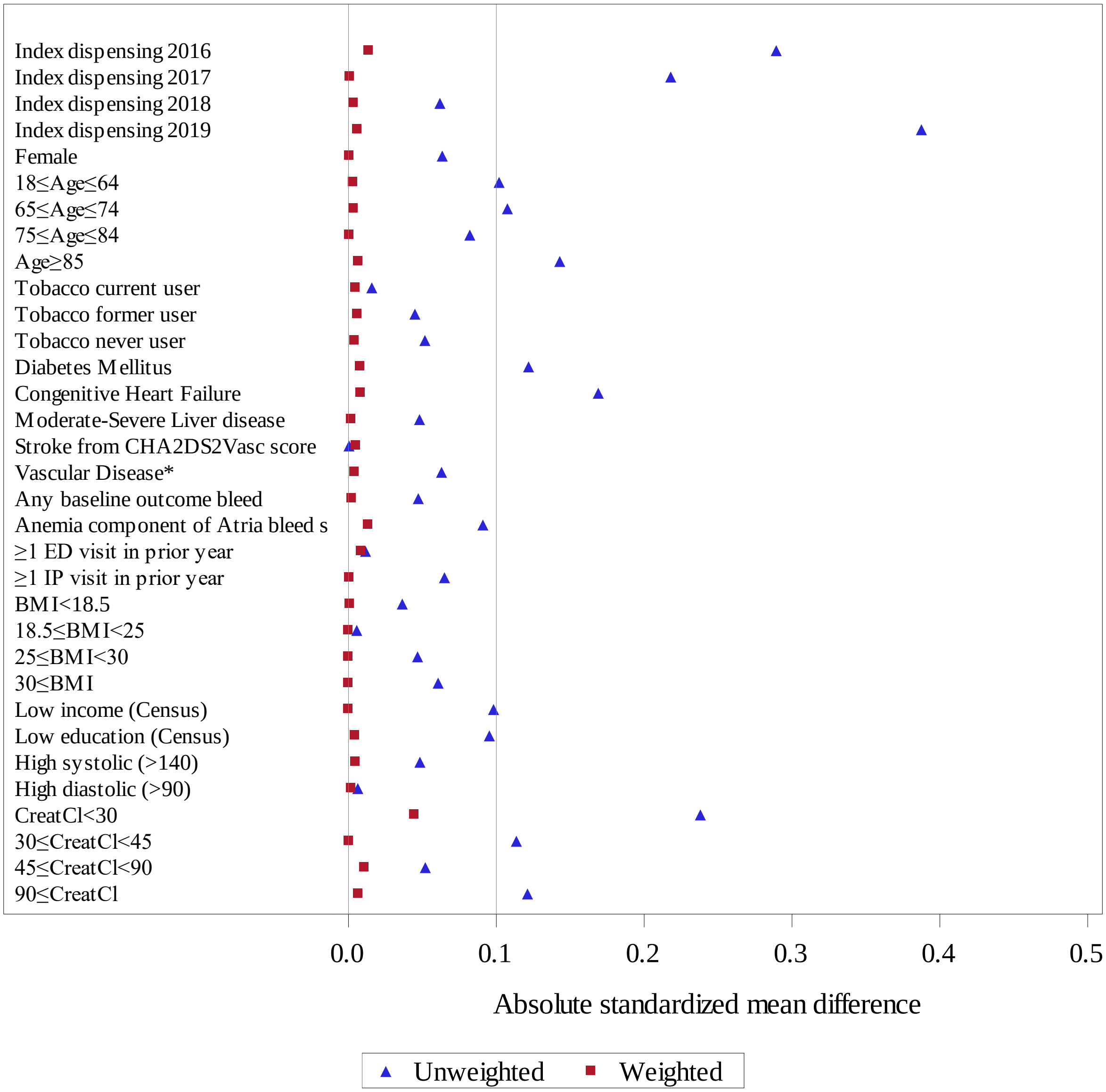

*Vascular Disease: MI/Peripheral Artery Disease/Aortic plaque

In all panels, blue triangles represent the ASMD between the DOAC and warfarin groups before weighting; red squares represent the ASMD after weighting. To be appropriate for comparisons, the ASMD for each characteristic should be <0.1. *Abbreviations:* AF: atrial fibrillation; ASMD: absolute standardized mean difference; BMI: body mass index; CreatCl: Creatinine Clearance; DOAC: direct oral anticoagulant; ED: emergency department; IP: inpatient; PMT: population management tool

© 2023 Derington CG et al. *JAMA Network Open.*

eFigure 5. Propensity Score Histograms for Receiving Care in a Specific DOAC Care Model Among DOAC Users (Post Hoc Analysis)

Panel A: Probability of receiving care in DOAC UC+PMT model vs. DOAC UC model

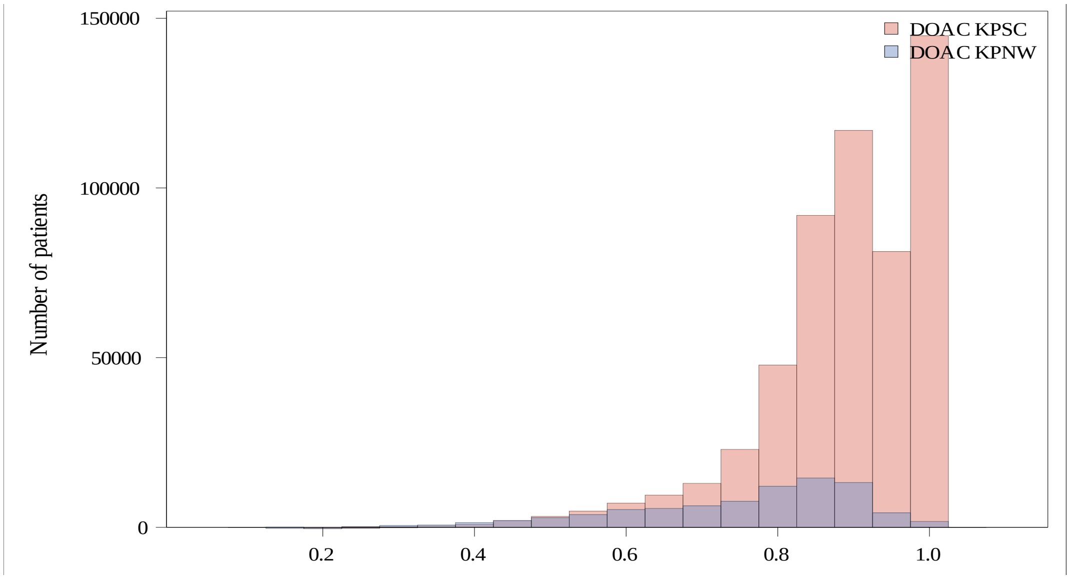

Panel B: Probability of receiving care in the DOAC AMS model vs. DOAC UC model

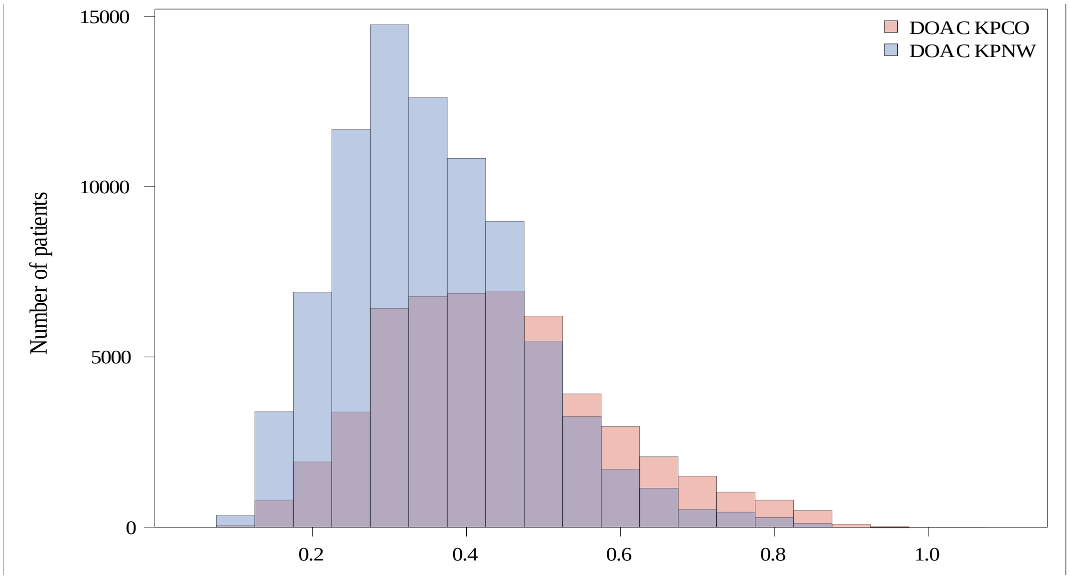

Abbreviations: AMS: anticoagulation management services; DOAC: direct oral anticoagulant; PMT: population management tool; UC: usual care

© 2023 Derington CG et al. *JAMA Network Open.*

eFigure 6. Balance of Patient Characteristics Before and After Inverse Probability of Treatment Weighting (Post Hoc Analysis)

Panel A: Receiving care in DOAC UC+PMT model vs. DOAC UC model

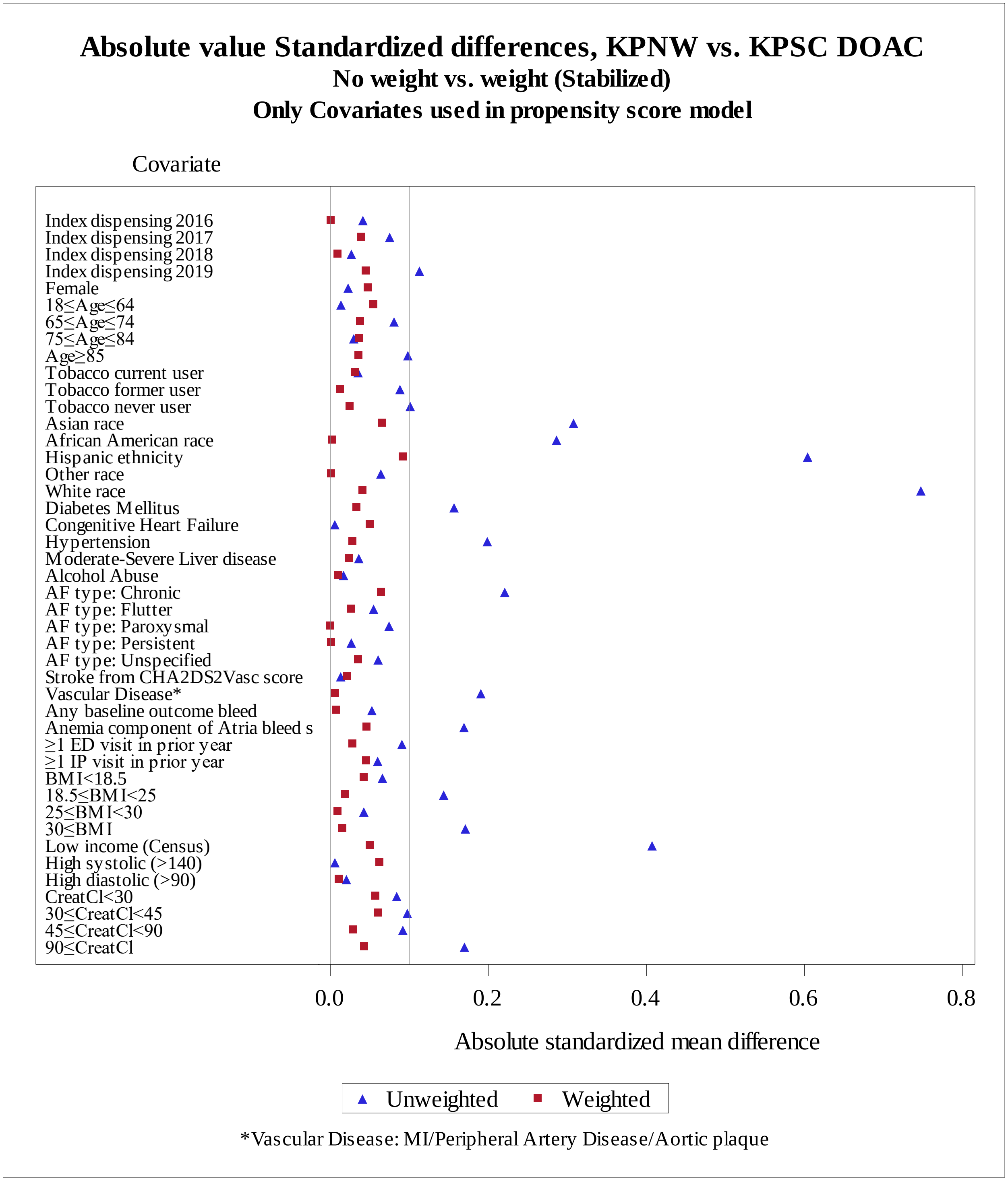

© 2023 Derington CG et al. *JAMA Network Open.*

Panel B: Receiving care in the DOAC AMS model vs. DOAC UC model

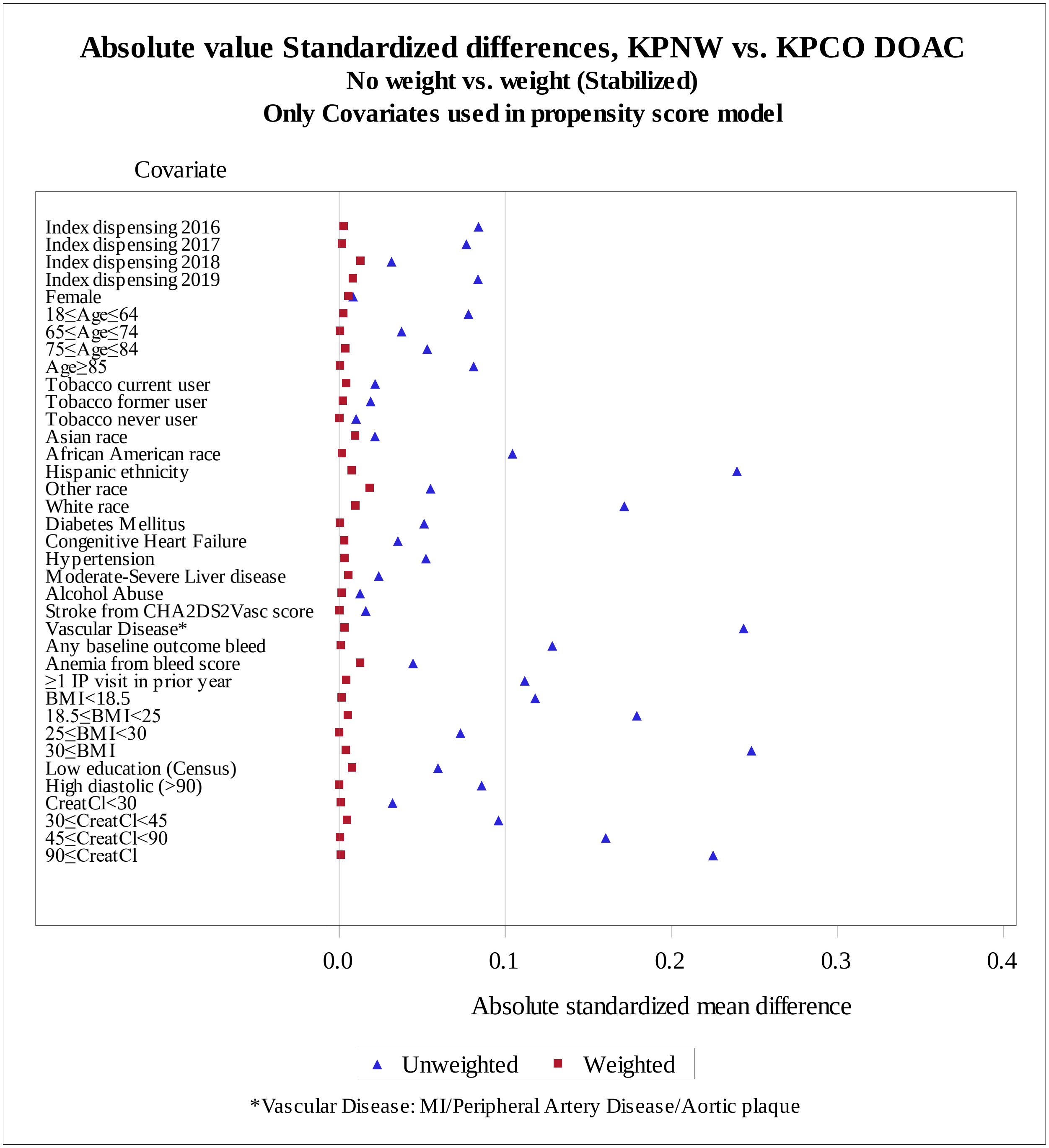

In all panels, blue triangles represent the ASMD between the DOAC and warfarin groups before weighting; red squares represent the ASMD after weighting. To be appropriate for comparisons, the ASMD for each characteristic should be <0.1. *Abbreviations*: AF: atrial fibrillation; ASMD: absolute standardized mean difference; BMI: body mass index; CreatCl: Creatinine Clearance; DOAC: direct oral anticoagulant; ED: emergency department; IP: inpatient; PMT: population management tool

© 2023 Derington CG et al. *JAMA Network Open.*

eFigure 7. Association of DOAC vs Warfarin Use and Bleeding Outcomes, by DOAC Management Model (a Priori Analysis)

Outcome and management model	DOAC No. of patients with an event/Total No. (%)	% per year	Warfarin No. of patients with an event/Total No. (%)	% per year	IP-Weighted Hazard Ratio (95% CI)
**Intracranial hemorrhage**					
UC	6/3297 (0.2)	0.09	29/2885 (1.0)	0.40	0.22 (0.08,0.56)
UC + PMT	134/21891 (0.6)	0.32	185/11734 (1.6)	0.66	0.50 (0.38,0.65)
AMS	5/2089 (0.2)	0.11	48/2850 (1.7)	0.70	0.19 (0.07,0.50)
**Gastrointestinal bleed**					
UC	45/3297 (1.4)	0.67	55/2885 (1.9)	0.77	1.21 (0.78,1.89)
UC + PMT	296/21891 (1.4)	0.71	277/11734 (2.4)	0.99	0.88 (0.72,1.08)
AMS	47/2089 (2.2)	1.06	71/2850 (2.5)	1.04	1.19 (0.81,1.76)
**Extracranial major bleed**					
UC	45/3297 (1.4)	0.67	61/2885 (2.1)	0.85	1.09 (0.71,1.68)
UC + PMT	296/21891 (1.4)	0.71	296/11734 (2.5)	1.06	0.79 (0.65,0.97)
AMS	48/2089 (2.3)	1.08	81/2850 (2.8)	1.19	1.03 (0.71,1.51)

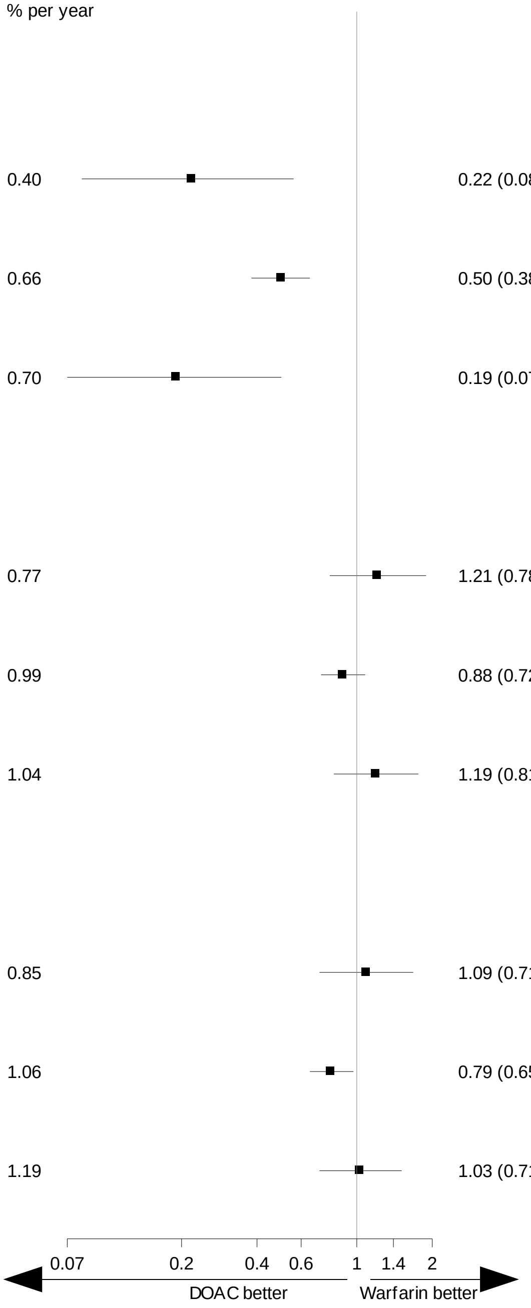

Number of events and percent with events per year are unweighted; hazard ratios are weighted by the inverse of the propensity score. *Composite endpoint of thromboembolic stroke, intracranial hemorrhage, gastrointestinal bleed, extracranial major bleed, or death.
AMS: anticoagulation management service; DOAC: direct oral anticoagulant; CI: confidence interval; IP: inverse probability; PMT: population management tool; UC: usual care

© 2023 Derington CG et al. *JAMA Network Open.*

eFigure 8. Medication Persistence Among DOAC Users

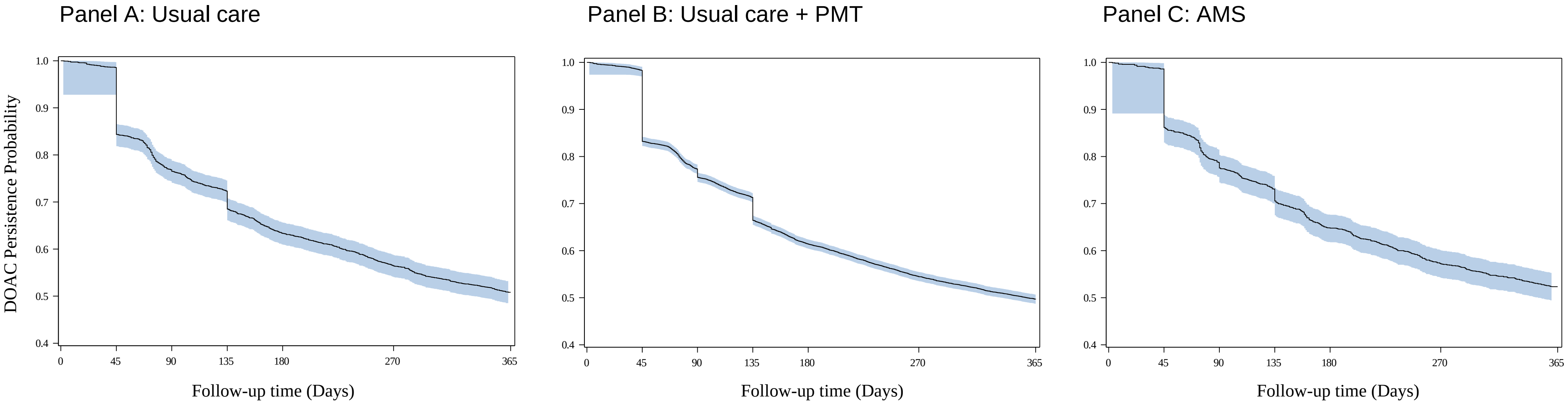

Abbreviations: AMS: anticoagulation management service; DOAC: direct oral anticoagulant; PMT: population management tool

© 2023 Derington CG et al. *JAMA Network Open.*

eFigure 9. Association of Incident DOAC vs Warfarin Use and the Composite Endpoint* in Subgroups

Subgroup and management model	DOAC No. of patients with an event/Total No. (%)	DOAC % per year	Warfarin No. of patients with an event/Total No. (%)	Warfarin % per year	IP-Weighted Hazard Ratio (95% CI)
**Age,years**					
**<65**					
UC	35/727 (4.8)	2.35	41/450 (9.1)	3.68	1.16 (0.71,1.90)
UC + PMT	217/4708 (4.6)	2.39	256/2056 (12.5)	5.31	0.91 (0.72,1.16)
AMS	15/395 (3.8)	1.82	37/430 (8.6)	3.63	0.77 (0.41,1.46)
**≥65 and <80**					
UC	155/1865 (8.3)	3.94	277/1560 (17.8)	6.98	0.83 (0.67,1.03)
UC + PMT	1048/11378 (9.2)	4.70	1168/5678 (20.6)	8.36	0.83 (0.74,0.92)
AMS	87/1144 (7.6)	3.37	214/1442 (14.8)	5.91	0.67 (0.51,0.86)
**≥80**					
UC	170/705 (24.1)	13.48	319/875 (36.5)	16.45	0.95 (0.77,1.17)
UC + PMT	1249/5805 (21.5)	12.72	1473/4000 (36.8)	16.95	0.88 (0.80,0.96)
AMS	121/550 (22.0)	12.28	283/978 (28.9)	14.01	1.03 (0.82,1.29)
**Sex**					
**Male**					
UC	180/1836 (9.8)	4.78	318/1563 (20.3)	8.18	0.94 (0.77,1.15)
UC + PMT	1347/12432 (10.8)	5.74	1537/6602 (23.3)	9.97	0.87 (0.79,0.95)
AMS	107/1172 (9.1)	4.29	273/1509 (18.1)	7.73	0.75 (0.59,0.94)
**Female**					
UC	180/1461 (12.3)	6.16	319/1322 (24.1)	10.17	0.86 (0.71,1.06)
UC + PMT	1167/9459 (12.3)	6.57	1360/5132 (26.5)	11.27	0.82 (0.75,0.91)
AMS	116/917 (12.6)	6.12	261/1341 (19.5)	8.34	0.95 (0.75,1.20)
**Race-ethnicity**					
**Non-Hispanic White**					
UC	325/3027 (10.7)	5.27	580/2604 (22.3)	9.13	0.87 (0.75,1.01)
UC + PMT	1588/13548 (11.7)	6.22	1677/6732 (24.9)	10.35	0.85 (0.78,0.93)
AMS	183/1776 (10.3)	4.81	442/2366 (18.7)	7.92	0.81 (0.68,0.97)
**All other race-ethnicities**					
UC	35/247 (14.2)	7.28	57/268 (21.3)	8.95	1.37 (0.86,2.18)
UC + PMT	871/7817 (11.1)	5.90	1187/4806 (24.7)	10.93	0.83 (0.74,0.92)
AMS	32/259 (12.4)	6.74	87/436 (20.0)	8.97	0.90 (0.59,1.39)
**Body weight, kg**					
**<60**					
UC	58/327 (17.7)	9.82	99/279 (35.5)	16.35	0.87 (0.61,1.25)
UC + PMT	480/2451 (19.6)	11.48	625/1599 (39.1)	18.97	0.79 (0.68,0.92)
AMS	54/265 (20.4)	11.22	120/398 (30.2)	14.61	0.91 (0.65,1.28)
**≥60**					
UC	302/2970 (10.2)	4.95	538/2606 (20.6)	8.38	0.89 (0.76,1.04)
UC + PMT	2034/19440 (10.5)	5.49	2272/10135 (22.4)	9.39	0.87 (0.81,0.94)
AMS	169/1824 (9.3)	4.32	414/2452 (16.9)	7.09	0.80 (0.66,0.97)
**Creatinine clearance, mL/min**					
**<30**					
UC	19/45 (42.2)	30.52	102/195 (52.3)	31.11	0.91 (0.48,1.75)
UC + PMT	211/549 (38.4)	29.78	716/1509 (47.5)	29.70	0.84 (0.69,1.02)
AMS	15/37 (40.5)	26.23	75/180 (41.7)	28.83	0.65 (0.34,1.27)
**≥30 and <50**					
UC	94/327 (28.7)	16.80	174/496 (35.1)	16.60	1.08 (0.82,1.42)
UC + PMT	630/2981 (21.1)	12.69	834/2413 (34.6)	15.34	0.88 (0.78,1.00)
AMS	66/296 (22.3)	12.37	145/513 (28.3)	13.22	1.10 (0.81,1.49)
**≥50**					
UC	230/2736 (8.4)	4.04	340/2068 (16.4)	6.37	0.83 (0.70,1.00)
UC + PMT	1625/17653 (9.2)	4.76	1305/7554 (17.3)	6.88	0.85 (0.78,0.93)
AMS	138/1698 (8.1)	3.75	305/2085 (14.6)	5.95	0.77 (0.62,0.94)
**CHADS2-Vasc score**					
**<2 (low stroke risk)**					
UC	8/462 (1.7)	0.77	13/193 (6.7)	2.58	0.50 (0.20,1.24)
UC + PMT	59/2406 (2.5)	1.22	25/738 (3.4)	1.29	0.70 (0.39,1.25)
AMS	3/294 (1.0)	0.45	8/221 (3.6)	1.45	0.36 (0.09,1.37)
**≥2 (intermediate/high stroke risk)**					
UC	352/2835 (12.4)	6.19	624/2692 (23.2)	9.57	0.91 (0.78,1.05)
UC + PMT	2455/19485 (12.6)	6.75	2872/10996 (26.1)	11.24	0.85 (0.79,0.91)
AMS	220/1795 (12.3)	5.91	526/2629 (20.0)	8.61	0.86 (0.73,1.01)
**ATRIA bleed score**					
**<4 (low risk)**					
UC	163/1960 (8.3)	3.96	244/1544 (15.8)	6.11	0.86 (0.69,1.06)
UC + PMT	1159/13885 (8.3)	4.29	1058/6288 (16.8)	6.55	0.83 (0.75,0.92)
AMS	103/1312 (7.9)	3.54	263/1765 (14.9)	6.01	0.75 (0.59,0.95)
**≥4 (intermediate/high risk)**					
UC	149/569 (26.2)	15.07	321/847 (37.9)	18.54	0.95 (0.77,1.18)
UC + PMT	1038/4098 (25.3)	15.86	1577/4108 (38.4)	19.98	0.89 (0.81,0.97)
AMS	95/345 (27.5)	16.59	223/682 (32.7)	17.75	1.07 (0.83,1.39)

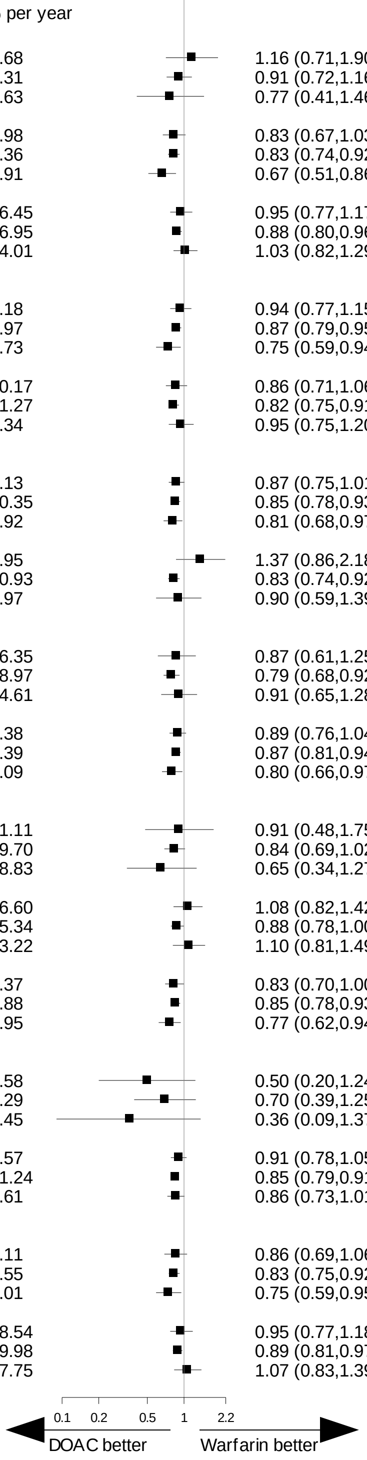

* Composite endpoint of thromboembolic stroke, intracranial hemorrhage, gastrointestinal bleed, extracranial major bleed, or death. AMS: anticoagulation management services; DOAC: direct

© 2023 Derington CG et al. *JAMA Network Open.*

oral anticoagulant; CI: confidence interval; IP: inverse propensity; PMT: population management tool; UC: usual care

© 2023 Derington CG et al. *JAMA Network Open.*

eFigure 10. Association of Incident DOAC vs Warfarin Use and the Composite Endpoint* by Sensitivity Analysis

Analysis, DOAC care model	DOAC users: No. of patients with an event/Total No. (%)	DOAC users: % per year	Warfarin users: No. of patients with an event/Total No. (%)	Warfarin users: % per year	IP-Weighted Hazard Ratio (95% CI)
**Primary anaysis**					
UC	360/3297 (10.9)	5.38	637/2885 (22.1)	9.07	0.91 (0.79,1.05)
UC + PMT	2514/21891 (11.5)	6.10	2897/11734 (24.7)	10.54	0.85 (0.79,0.90)
AMS	223/2089 (10.7)	5.08	534/2850 (18.7)	8.02	0.84 (0.72,0.99)
**Complete case analysis**					
UC	298/2420 (12.3)	6.09	551/2315 (23.8)	9.94	0.91 (0.78,1.06)
UC + PMT	2114/17139 (12.3)	6.60	2572/10080 (25.5)	11.02	0.85 (0.80,0.92)
AMS	195/1587 (12.3)	5.85	473/2356 (20.1)	8.74	0.88 (0.74,1.05)
**Exclude patients with prior event**					
UC	312/3102 (10.1)	4.92	570/2704 (21.1)	8.60	0.89 (0.76,1.03)
UC + PMT	2249/20706 (10.9)	5.73	2692/11159 (24.1)	10.20	0.84 (0.78,0.90)
AMS	184/1909 (9.6)	4.54	468/2621 (17.9)	7.53	0.82 (0.68,0.98)
**Early outcome events only**					
UC	104/3297 (3.2)	12.95	140/2885 (4.9)	20.08	0.97 (0.73,1.28)
UC + PMT	838/21891 (3.8)	15.79	678/11734 (5.8)	24.06	0.94 (0.83,1.07)
AMS	79/2089 (3.8)	15.62	146/2850 (5.1)	21.32	0.94 (0.70,1.25)
**Late outcome events only**					
UC	256/3147 (8.1)	3.84	497/2730 (18.2)	7.09	0.88 (0.75,1.04)
UC + PMT	1676/20702 (8.1)	4.08	2219/10931 (20.3)	8.10	0.82 (0.76,0.89)
AMS	144/1982 (7.3)	3.29	388/2668 (14.5)	5.84	0.81 (0.67,0.99)
**Follow up through 12/31/2019**					
UC	307/3297 (9.3)	7.63	577/2885 (20.0)	11.59	0.94 (0.81,1.09)
UC + PMT	2273/21891 (10.4)	7.64	2737/11734 (23.3)	12.15	0.87 (0.81,0.93)
AMS	219/2089 (10.5)	7.92	526/2850 (18.5)	11.18	0.87 (0.74,1.02)

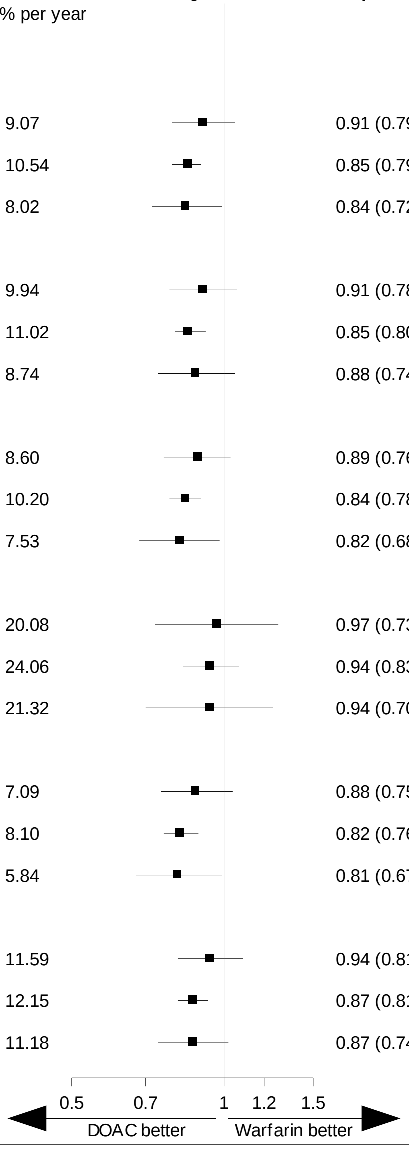

Number of events and percent with events per year are unweighted. * Composite endpoint of thromboembolic stroke, intracranial hemorrhage, gastrointestinal bleed, extracranial major bleed, or death. See eMethods for more detailed descriptions of each sensitivity analysis. AMS: anticoagulation management services; DOAC: direct oral anticoagulant; CI: confidence interval; IP: inverse propensity; PMT: population management tool; UC: usual care

© 2023 Derington CG et al. *JAMA Network Open.*

eReferences.

1. Hawkins KL, King JB, Delate T, Martinez K, McCool K, Clark NP. Pharmacy Technician Management of Stable, In-Range INRs Within a Clinical Pharmacy Anticoagulation Service. *J Manag Care Spec Pharm*. 2018;24(11):1130-1137. doi:10.18553/jmcp.2018.24.11.1130
2. Shore S, Ho PM, Lambert-Kerzener A, et al. Site-level variation in and practices associated with dabigatran adherence. *JAMA*. 2015;313(14):1443-1450. doi:10.1001/jama.2015.2761
3. Paquette M, Mbuagbaw L, Iorio A, Nieuwlaat R. Methodological considerations for investigating oral anticoagulation persistence in atrial fibrillation. . *European Journal of Cardiovascular Pharmacotherapy*. 2021;7(3):251-260. doi:10.1093/ehjcvp/pvaa052
4. Sikka R, Xia F, Aubert RE. Estimating medication persistency using administrative claims data. *Am J Manag Care*. 2005;11(7):449-457.
5. Lip G, Nieuwlaat R, Pisters R, Lane DA, Crinjs H. Refining clinical risk stratification for predicting stroke and thromboembolism in atrial fibrillation using a novel risk factor-based approach: the euro heart survey on atrial fibrillation. *Chest*. 2010;137(2):263-272. doi:10.1378/chest.09-1584
6. Friberg L, Rosenqvist M, Lip G. Evaluation of risk stratification schemes for ischaemic stroke and bleeding in 182 678 patients with atrial fibrillation: The Swedish Atrial Fibrillation cohort study. *European Heart Journal*. 2012;33(12):1500-1510. doi:10.1093/eurheartj/ehr488
7. Fang MC, Go AS, Chang YC, et al. A New Risk Scheme to Predict Warfarin-Associated Hemorrhage: The ATRIA (Anticoagulation and Risk Factors in Atrial Fibrillation) Study. *Journal of the American College of Cardiology*. 2011;58(4):395-401. doi:10.1016/j.jacc.2011.03.031
8. Afzal N, Mallipeddi VP, Sohn S, et al. Natural language processing of clinical notes for identification of critical limb ischemia. International Journal of Medical Informatics: Elsevier; 2018. p. 83-89.
9. Brookhart A, Schneeweiss S, Rothman KJ, Glynn RJ, Avorn J, Sturmer T. Variable selection for propensity score models. *American Journal of Epidemiology*. 2006;163(12):1149-1156. doi:10.1093/aje.kwj149
10. Austin PC. Variance estimation when using inverse probability of treatment weighting (IPTW) with survival analysis. *Stat Med*. 2016;35(30):5642-5655. doi:10.1002/sim.7084
11. Li L, Greene T. A weighting analogue to pair matching in propensity score analysis. International Journal of Biostatistics2013. p. 215-234.
12. Initiative S. Thromboembolic Stroke, Intracranial Hemorrhage, Gastrointestinal Bleeding, and Major Extracranial Bleeding following Dabigatran, Rivaroxaban, and Apixaban Use in Patients with Atrial Fibrillation: A Propensity Score Matched Analysis. Food and Drug Administration, Center for Drug Evaluation and Research. 03-28, 2022. Accessed 11-11, 2019. https://www.sentinelinitiative.org/studies/drugs/individual-drug-queries/thromboembolic-stroke-intracranial-hemorrhage
13. Go AS, Singer DE, Toh S, et al. Outcomes of Dabigatran and Warfarin for Atrial Fibrillation in Contemporary Practice. *Annals of Internal Medicine*. 2017;167(12):845-854. doi:10.7326/M16-1157

© 2023 Derington CG et al. *JAMA Network Open.*
