# Supplementary material for: Association of Direct Oral Anticoagulation Management Strategies With Clinical Outcomes for Adults With Atrial Fibrillation
Source: JAMA Netw Open. 2023 Jul 6;6(7):e2321971. doi: 10.1001/jamanetworkopen.2023.21971 (PMC10326649; doi:10.1001/jamanetworkopen.2023.21971)
Supplement: Supplement 2. — Data Sharing Statement [file jamanetwopen-e2321971-s002.pdf]

Data Sharing Statement

Derington. Association of Direct Oral Anticoagulation Management Strategies With Clinical Outcomes for Adults With Atrial Fibrillation. *JAMA Netw Open*. Published July 06, 2023. doi:10.1001/jamanetworkopen.2023.21971

Data
Data available: No
